# High-Order Epistasis in Catalytic Power of Dihydrofolate Reductase Gives Rise to a Rugged Fitness Landscape in the Presence of Trimethoprim Selection

**DOI:** 10.1093/molbev/msz086

**Published:** 2019-04-15

**Authors:** Yusuf Talha Tamer, Ilona K Gaszek, Haleh Abdizadeh, Tugce Altinusak Batur, Kimberly A Reynolds, Ali Rana Atilgan, Canan Atilgan, Erdal Toprak

**Affiliations:** 1 Green Center for Systems Biology, University of Texas Southwestern Medical Center, Dallas, TX; 2 Faculty of Science and Engineering, University of Groningen, Groningen, Netherlands; 3 Faculty of Engineering and Natural Sciences, Sabanci University, Istanbul, Turkey; 4 Department of Internal Medicine, Dokuz Eylul University, Izmir, Turkey; 5 Department of Biophysics, University of Texas Southwestern Medical Center, Dallas, TX; 6 Department of Pharmacology, University of Texas Southwestern Medical Center, Dallas, TX

**Keywords:** antibiotic resistance, molecular evolution, experimental evolution, epistasis, protein evolution

## Abstract

Evolutionary fitness landscapes of several antibiotic target proteins have been comprehensively mapped showing strong high-order epistasis between mutations, but understanding these effects at the biochemical and structural levels remained open. Here, we carried out an extensive experimental and computational study to quantitatively understand the evolutionary dynamics of *Escherichia coli* dihydrofolate reductase (DHFR) enzyme in the presence of trimethoprim-induced selection. To facilitate this, we developed a new in vitro assay for rapidly characterizing DHFR steady-state kinetics. Biochemical and structural characterization of resistance-conferring mutations targeting a total of ten residues spanning the substrate binding pocket of DHFR revealed distinct changes in the catalytic efficiencies of mutated DHFR enzymes. Next, we measured biochemical parameters (*K*_m_, *K_i_*, and *k*_cat_) for a mutant library carrying all possible combinations of six resistance-conferring DHFR mutations and quantified epistatic interactions between them. We found that the high-order epistasis in catalytic power of DHFR (*k*_cat_ and *K*_m_) creates a rugged fitness landscape under trimethoprim selection. Taken together, our data provide a concrete illustration of how epistatic coupling at the level of biochemical parameters can give rise to complex fitness landscapes, and suggest new strategies for developing mutant specific inhibitors.

## Introduction

Antibiotic resistance is one of the most important global health threats (Laxminarayan et al. 2013). According to the Centers for Disease Control and Prevention, antibiotic resistant pathogens cause over 20,000 deaths and 2 million infections annually in the United States alone (CDC 2013). Antibiotic resistance evolves either by resistance-conferring spontaneous mutations in bacterial genomes or horizontal transfer of mobile resistance elements (Martinez 2008; Davies and Davies 2010). These genetic changes typically confer resistance by reducing the affinities of antibiotic molecules to their targets, deactivating antibiotics by chemical modification, and finally decreasing effective antibiotic concentrations inside bacterial cytoplasm by either efflux pumps or reduced uptake of antibiotic molecules (Blair et al. 2015). Among these, understanding how mutations render antibiotics ineffective by altering their targets is particularly important from both clinical and basic science perspectives (Weinreich et al. 2006; Rodrigues et al. 2016).

In pathogenic bacteria, there is only a handful of drug target proteins, such as DNA gyrases and RNA polymerases and finding new “druggable” enzymes or novel drugs that can target resistant bacteria is often a long and extremely difficult process (Smith and Calvo 1982; Huovinen et al. 1995; Xu et al. 1996; Comas et al. 2012; Hartkoorn et al. 2012). Mutations in these target proteins such as InhA (Enoyl-ACP reductase), RNA polymerases, dihydrofolate reductase (DHFR), GyrB (DNA gyrase subunit B), and the ribosomal protein RpsL are known to render several important antibiotics ineffective. Therefore, a mechanistic understanding of resistance-conferring mutations in antibiotic targets is critical for designing new drugs or drug variants that can inhibit antibiotic resistant bacteria (Dasgupta et al. 2009; Pokrovskaya et al. 2009). How essential enzymes can preserve their catalytic activities when they acquire mutations to reduce drug affinity is another important question for better understanding basic principles driving protein evolution (Salverda et al. 2010; Palmer et al. 2015; Schenk et al. 2015; Stiffler et al. 2015; Rodrigues et al. 2016). In this study, we scrutinize molecular mechanisms of resistance-conferring mutations in the *Escherichia coli* DHFR enzyme and investigate how epistasis between these mutations shapes the adaptive landscape for trimethoprim (TMP) resistance evolution.

**Fig. 1.**
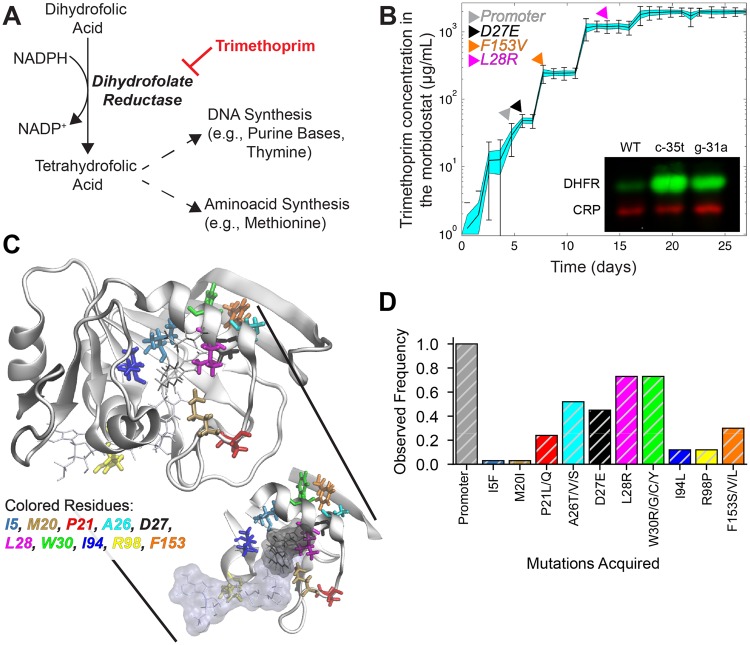
TMP resistance evolves through sequential accumulation of DHFR mutations. (*A*) Enzymatic activity of DHFR is crucial for nucleotide and amino acid synthesis in *Escherichia coli*. TMP is a competitive inhibitor of DHFR that blocks its enzymatic activity by occupying its active site. (*B*) TMP dose fluctuations (cyan filled area) in a morbidostat experiment are shown. Solid black line centering the cyan area represent mean TMP concentrations, error bars represent daily standard deviations of TMP concentrations, and width of the cyan area reflect the standard error of the mean of TMP concentrations at a given time. Morbidostat experiments revealed stepwise acquisition of resistance-conferring mutations; a sample morbidostat trajectory demonstrating temporal changes in TMP resistance. Colored arrows indicate the timing of the first detection of DHFR mutations. (Insert) Promoter mutations (c-35t, g-31a) lead to 10- to 20-fold higher DHFR protein amount relative to WT. (*C*) Mutated DHFR residues are highlighted in different colors on DHFR structure (PDB ID: 1rx2). (*D*) Observed frequencies of resistance-conferring mutations plotted for 33 independent morbidostat experiments (28 populations from this study and 5 populations from a previous study [Toprak et al. 2011]).

DHFR is a ubiquitous enzyme in nature with an essential role in folic acid synthesis (Matthews et al. 1977; Benkovic et al. 1988; Schnell et al. 2004). Due to its central role in metabolism (fig. 1*A*), DHFR is used as a drug target in antibacterial, anticancer, antirheumatic, and antimalarial therapies (Schnell et al. 2004). For instance, pyrimethamine is one of the few available drugs that can be used for treating malaria caused by *Plasmodium falciparum*. Pyrimethamine has specific toxicity against *P. falciparum* by binding and inhibiting the *P. falciparum* dihydrofolate reductase (pfDHFR) enzyme (Dasgupta et al. 2009; Lozovsky et al. 2009; Yuthavong et al. 2012). However, although pyrimethamine was one of the most commonly used drugs for malaria treatment in the past, as of today, it is rarely prescribed because of the resistance problem (Lozovsky et al. 2009; Hecht and Fogel 2012). The most common resistance-conferring mutations in pfDHFR are the four point mutations N51I, C59R, S108N, and I164L (Lozovsky et al. 2009; Yuthavong et al. 2012). The quadruple mutant of pfDHFR that carries all four of these mutations is widespread globally and is highly resistant to pyrimethamine. Similarly, evolution of resistance to TMP, a bacteriostatic antibiotic molecule that competitively binds to DHFR and blocks its enzymatic activity, proceeds through sequential accumulation of resistance-conferring mutations in the bacterial DHFR enzyme (Toprak et al. 2011; Oz et al. 2014). In our previous work, we showed that *E. coli* cells evolved TMP resistance by accumulating up to four DHFR mutations in a stepwise fashion (Toprak et al. 2011; Oz et al. 2014; Palmer et al. 2015). Since DHFR is an essential enzyme, the evolution of resistance against DHFR inhibiting drugs is a search for finding DHFR mutants that have reduced drug affinity and yet adequate catalytic power for organismal survival. For better understanding the evolutionary dynamics of resistance against DHFR inhibitors, it is important to quantitatively evaluate evolutionary paths leading to antibiotic resistance and characterize resistance at the enzyme structure level for the ultimate goal of improving human health.

We carried out a comprehensive experimental and computational study to better understand the evolutionary dynamics of *E. coli* DHFR in the presence of TMP. In the following part of this text, DHFR will be used to refer *E. coli* DHFR enzyme. We evolved several antibiotic-naïve *E. coli* populations against TMP in the morbidostat, a continuous culture device we developed to quantitatively study evolution of antibiotic resistance (Toprak et al. 2011, 2013). We then identified genetic changes in *E. coli* that were responsible for TMP resistance. The genetic changes we found were mostly in the *folA* gene that encodes for DHFR. We identified ten residues that were frequently mutated in the DHFR as well as promoter mutations that significantly increased DHFR protein levels in bacteria. We developed a new biochemical assay that enabled us rapidly characterize these mutations by quantifying their effects on substrate binding (*K*_m_), and catalytic rate (*k*_cat_) of DHFR. We synthesized all possible combinations for six of these DHFR mutations and quantified epistatic interactions between these mutations. Finally, we measured the effects of these mutations on bacterial fitness by replacing the endogenous *folA* gene in *E. coli* with its mutated variants. Our analyses show that the adaptive landscape of DHFR, calculated using biochemical properties of DHFR mutants, deviates from the landscape predicted from the fitness effects of single DHFR mutations using an independence model, where fitness effects of multiple mutations are assumed to be additive (Tekin et al. 2018). We show that this deviation is mainly because of the high-order epistasis between mutations altering DHFR catalytic activity and substrate binding. Next, by running computer simulations, we identified plausible genetic trajectories that reach to TMP-resistant genotypes. Our simulations suggest that the evolution of TMP resistance can be impeded by exploiting epistatic interactions between resistance-conferring mutations and the use of mutant specific inhibitors. Finally, we carried out molecular dynamics (MD) simulations to reveal structural changes responsible for TMP resistance and epistatic interactions between mutations. Analysis of the MD simulations suggests that DHFR mutations confer resistance by utilizing distinct structural changes which may be exploited for drug design purposes.

## Results

DHFR catalyzes the reduction of 7,8-dihydrofolate (DHF) to 5,6,7,8-tetrahydrofolate (THF) by hydride transfer from nicotinamide adenine dinucleotide phosphate (NADPH) (fig. 1*A*) (Benkovic et al. 1988; Huennekens 1996; Hammes-Schiffer 2004; Schnell et al. 2004; Boehr et al. 2006, 2008). THF is an essential precursor for cell growth as it is used in thymidylate and purine synthesis. Therefore, inhibition of bacterial DHFR slows down or stops bacterial growth. TMP is a bacterial DHFR inhibitor which competitively binds to the active site of DHFR (fig. 1*A*). It is a commonly used antibiotic compound for treating bacterial infections and is typically used in combination with sulfamethoxazole due to synergism in their combined effects. We and others have previously run laboratory evolution experiments to explore evolutionary trajectories that lead to high levels of TMP resistance in *E. coli* (Toprak et al. 2011; Oz et al. 2014; Baym et al. 2016). In these studies, it was shown that TMP resistance evolved in a stepwise fashion and all populations acquired multiple mutations in the *folA* gene that encodes DHFR. This observation was consistent with previous studies reporting multiple DHFR mutations in clinically isolated TMP-resistant pathogens (Maskell et al. 2001; Queener et al. 2013). One of the resistance-conferring mutations was always in the promoter region and the rest were in the coding region of *folA*. Mutations elsewhere in the genome were rare implying that the evolution of TMP resistance was confined to a small genetic target (Toprak et al. 2011). Although our results suggested a reproducibility in the temporal order of the DHFR mutations, the number of evolved populations was small and it was not clear whether the mutations we observed were covering all possible DHFR mutations. Besides, since a decrease in DHFR’s catalytic efficiency is expected to decrease bacterial fitness (Reynolds et al. 2011), it was not clear whether evolutionary trajectories would have been different if the minimum allowed growth rate in an evolution experiment was changed.

### *Escherichia coli* Populations Evolving under Mild TMP Selection Follow Less-Constrained Mutational Trajectories

We evolved 28 initially isogenic and TMP-sensitive *E. coli* populations in the morbidostat using different minimum growth rate constraints (Toprak et al. 2011, 2013). Morbidostat is an automated continuous culture device that maintains nearly constant selection pressure throughout the evolution experiment. This is achieved by continuously monitoring bacterial growth and clamping bacterial growth rate by adjusting antibiotic concentrations with the help of computer-controlled pumps. Addition of plain growth media or antibiotic containing growth media is periodically done at constant dilution rates. Therefore, bacterial populations that cannot grow faster than the dilution rate of the morbidostat are washed out and hence cannot survive in the morbidostat. This feature enabled us run evolution experiments at different dilution settings and controls the minimal growth rate allowed for the survival of bacterial populations. In our settings, the drug-free exponential growth rate of the parental *E. coli* strain (MG1655) was ∼0.8 h^−1^ (doubling time = ∼52 min; M9 minimal media supplemented with casamino acids and glucose, at ∼30 °C). We evolved initially isogenic and antibiotic-naïve *E. coli* populations (Materials and Methods) at two different dilution rates (0.3 h^−1^ [mild selection, *n *=* *14] and 0.6 h^−1^ [strong selection, *n *=* *14]) for several weeks and asked whether there would be any difference in the dynamics of TMP resistance evolution. Selection is stronger in the settings where the dilution rate is adjusted to be 0.6 h^−1^ as bacterial populations cannot survive in the morbidostat if they have doubling times longer than ∼70 min, whereas under mild selection, populations can survive in the morbidostat if bacterial cells can double every 140 min or faster.

All *E. coli* populations in the morbidostat evolved very high TMP resistance in a stepwise fashion (fig. 1*B* and supplementary fig. S1, Supplementary Material online) and they were able to survive even at ∼2.5 mg/ml TMP concentration which is the maximum solubility limit of TMP in our growth media (M9 minimal media supplemented with casamino acids and glucose, at 30 °C). All of the populations acquired two to four mutations in the *folA* gene (supplementary fig. S1, Supplementary Material online) and whole genome sequencing of 13 randomly selected TMP-resistant mutants that were isolated on the last day of morbidostat experiments revealed that, although the *folA* gene with its regulatory region spans only ∼0.013% of the genome, 39 out of 73 mutations (53%) were observed in *folA* (supplementary table S1, Supplementary Material online). One of the *folA* mutations was always a promoter mutation (g-9a, c-15a, g-31a, or c-35t) and these promoter mutations increased DHFR protein expression levels 10–20 times compared with their wild-type (WT) ancestor (fig. 1*B*, insert). The rest of the *folA* mutations were in the coding region of *folA* and targeted total of ten residues spanning the substrate binding pocket as illustrated in figure 1*C*. Among these, the most common mutations were at the following residues: P21, A26, D27, L28, W30, and F153 (fig. 1*D*). When we closely examined population structures and mutational trajectories of the evolving bacterial cultures, although there were no significant difference in the final resistance level between the cultures evolved under strong selection and mild selection, we found that *E. coli* cells evolved under mild selection experienced softer sweeps and acquired more *folA* mutations compared with *E. coli* cells evolved under strong selection (supplementary fig. S1, Supplementary Material online, ∼3.5 ± 0.5 mutations vs. ∼3.07 ± 0.46 mutations, *P *<* *0.01, Student’s *t*-test). Besides, we found that populations evolved under strong selection went through harder sweeps and had relatively less diversity within the resistance-conferring *folA* mutations (supplementary fig. S1, Supplementary Material online). Particularly, under strong selection, the first mutation in the coding region of *folA* was dominantly the L28R mutation (9 times out of 14). However, in the case of mild selection, the early mutations in the coding region of *folA* showed more variation (supplementary fig. S1, Supplementary Material online). This observation suggested that under strong selection, evolving populations were more constrained while acquiring resistance-conferring DHFR mutations.

### Resistance-Conferring Mutations Have Diverse Effects on Catalytic Efficiency of DHFR

**Fig. 2.**
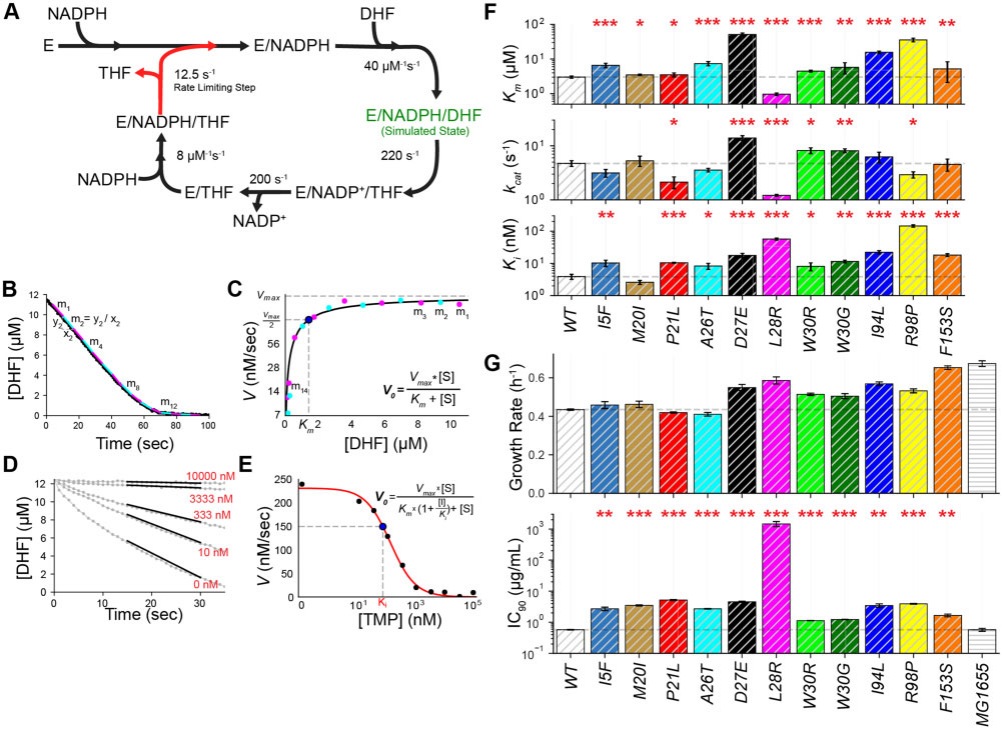
Biochemical characterization of resistance-conferring DHFR mutations. (*A*) Catalytic cycle of DHFR. Forward reaction rates are obtained from Schnell et al. (2004). Rate-limiting step in the catalytic cycle is release of THF (red arrow). E stands for DHFR. E-NADPH-DHF (green fonts) is the state used in our MD simulations. (*B*) Left panel shows a typical reaction progression curve after absorbance (340 nm) values are converted to DHF concentration (see Materials and Methods). By utilizing moving time windows, we calculate catalysis rates at corresponding DHF concentrations. (*C*) *K*_m_ and *k*_cat_ values are predicted by fitting a Michelis–Menten equation to measured catalysis rates. (*D*, *E*) Initial reaction rates in the presence of various TMP concentrations are used to predict the affinity (*K*_i_) of DHFR mutants to TMP molecules. (*F*) *K*_m_, *k*_cat_, and *K*_i_ values of DHFR mutants with single amino acid replacements. Error bars show standard error of the mean. Student’s *t*-test (two tailed) is used to quantify significance of *K*_m_, *k*_cat_, and *K*_i_ changes relative to the WT DHFR (*P < 0.05, **P < 0.01, and ***P < 0.001). (*G*) (upper panel) All engineered *Escherichia coli* strains carrying single DHFR mutations are viable. Endogenous *folA* gene was replaced with the WT or mutated *folA* genes (Materials and Methods). Cells were grown at ∼30 °C in minimal M9 media supplemented with 0.4% glucose and 0.2% amicase in 12 replicates. Exponential growth rates of all mutants except the I5F and L28R are all significantly lower than the parental MG1655 *E. coli* strain but higher that the strain (WT) we engineered by reinserting the WT folA gene. Despite our several attempts, the engineered WT strain had a growth defect most likely as a result of the selection markers we used for cloning (Materials and Methods). (lower panel) All engineered *E. coli* strains carrying single DHFR mutations have elevated TMP resistance. Inhibitory concentrations reducing growth by 90% (IC_90_) were measured by growing mutants in a gradient of TMP using 12 replicates (∼30 °C in minimal M9 media supplemented with 0.4% glucose and 0.2% amicase). Student’s *t*-test (two tailed) is used to quantify significance of IC_90_ changes relative to the WT DHFR (*P < 0.05, **P < 0.01, and ***P < 0.001, error bars shows the standard error on the mean for each mutant).

Ideally, fitness effects of mutations should be measured at the organismal level. However, characterizing the evolutionary fitness landscape for DHFR requires reliable fitness measurements which are not always possible when in vivo assays are utilized. First, in our experience, several of the bacterial mutants carrying DHFR mutations survived even at the highest possible TMP concentrations we could achieve (∼2.5 mg/ml) making it impossible to measure their true resistance levels (Palmer et al. 2015). Second, despite our numerous attempts, it was not possible to engineer some of the *E. coli* strains with desired combinations of DHFR mutations, suggesting that cells with some *DHFR* alleles are not viable (Palmer et al. 2015). Third, the strain we engineered by replacing the endogenous *folA* (the gene that is transcribed into DHFR) with the WT *folA* gene had a growth defect compared with its ancestor MG1655 strain making growth rate measurements less reliable (fig. 2). Fourth, overexpression of DHFR due to promoter mutations masked the true fitness effects of mutations found in the coding region of *DHFR* (Palmer et al. 2015). Finally, it is generally difficult to unequivocally attribute the effects of mutations to bacterial fitness as cells can compensate deleterious effects of mutations by gene regulation or rearranging metabolic fluxes. Therefore, we decided to characterize fitness effects of DHFR mutations at the protein level by utilizing in vitro assays. A total of eighteen resistance-conferring mutations (spanning ten residues) in the coding region of the *folA* gene were detected. We studied eleven of these single mutations by choosing the most frequently observed amino acid replacement in each residue, except W30 where we studied both W30G and W30R mutations.

We developed a rapid in vitro assay for calculating *k*_cat_ and *K*_m_ values for mutated DHFR enzymes (fig. 2 and supplementary figs. S2 and S3, Supplementary Material online). Measuring substrate affinity (*K*_m_) and catalytic rate (*k*_cat_) of an enzyme typically requires enzymatic activity measurements at various substrate concentrations and predicting *k*_cat_ and *K*_m_ values by fitting a Michelis–Menten function to the resulting data (Reynolds et al. 2011; Bershtein et al. 2012; Rodrigues et al. 2016). Depending on the enzyme, this can be a laborious and expensive task. The standard assay used for measuring DHFR activity benefits from spectroscopic changes in the cofactor (NADPH) and substrate (DHF) of DHFR as THF is produced. Typically, by maintaining a high concentration of NADPH compared with the DHF, initial reduction rate of DHFR is calculated by monitoring the absorbance of NADPH and DHF at 340-nm wavelength. NADPH and DHF have high absorptions at 340 nm (*A*_340_) but their absorptions drop upon hydride transfer between them. When DHFR is mixed with NADPH and DHF, *A*_340_ is rapidly reduced until DHF is completely consumed; this measurement needs to be repeated at several different substrate concentrations for predicting *k*_cat_ and *K*_m_ values. We realized that this laborious assay was not necessary for characterizing DHFR activity. In the presence of saturating concentrations of DHF (10–20 µM) and NADPH (100–200 µM), DHFR molecules already sample all possible concentrations of DHF throughout the progression of the reaction while NADPH levels are still at saturating levels. Also, the spectroscopic properties of NADPH and DHF allow us to predict both DHF and NADPH concentrations during the progression of this reaction (supplementary fig. S2, Supplementary Material online). Since the rates of reverse reactions (fig. 2*A*, counterclockwise direction) in the catalytic cycle are very slow relative to the forward reaction rates (fig. 2*A*, clockwise direction), it is possible to calculate reaction rates at various DHF concentrations from a single reaction progression curve. As shown in figure 2*B* and supplementary figure S2, Supplementary Material online, we split the progression curve in equal time windows and calculate corresponding mean DHF concentrations and DHF reduction rates for every time interval. We then use these values to predict *k*_cat_ and *K*_m_ values by fitting a Michelis–Menten equation (fig. 2*C* and supplementary fig. S2, Supplementary Material online). The *k*_cat_ and *K*_m_ values we measured using this practical method correlated well (*r *=* *0.98 and *P *<* *10^−3^ for *k*_cat_; *r *=* *0.98 and *P *<* *10^−3^ for *K*_m_; Pearson correlation test) with the values we measured using the conventional method that needs measurements at several different DHF concentrations (supplementary fig. S3 and table S2, Supplementary Material online). In addition, by measuring DHFR activity at steady state using various TMP concentrations (fig. 2*D*), we calculated TMP affinities of DHFR mutants (*K*_i_) assuming competitive binding kinetics between DHF and TMP (fig. 2*E* and eq. 1) (Nelson and Cox 2008).

(1) $$V_{\left[\mathrm{TMP}\right]}=\frac{k_{\mathrm{cat}}.\left[\mathrm{DHFR}\right].\left[\mathrm{DHF}\right]}{K_{m}\;\left(1+\frac{\left[\mathrm{TMP}\right]}{K_{i}}+\frac{\left[\mathrm{DHF}\right]}{K_{m}}\right)}\;\mathrm{at}\;\mathrm{DHF}=12.5\;µM.$$

All of the mutations except the L28R caused significant reductions in the substrate affinity (increased *K*_m_) of DHFR (fig. 2*F* and supplementary table S3, Supplementary Material online). Contrary to our expectations, substrate affinity of the L28R mutant was significantly increased (lower *K*_m_) relative to the WT DHFR. Changes in the *K*_m_ were generally accompanied with significant changes in the *k*_cat_ values. Interestingly, three of the mutants (P21L, L28R, and R98P) exhibited decreased catalytic rates whereas others (D27E, W30G, and W30R) had increased catalytic rates *k*_cat_. Finally, all of the mutations but one (M20I) had reduced TMP affinity (increased *K*_i_). Although antibiotic resistance via target modifications is typically attributed to reduced drug and substrate affinities due to mutations, our measurements summarized in figure 2*F* suggest that there could be distinct resistance mechanisms. That being said, *K*_i_ values alone are far from enough for explaining TMP resistance (supplementary fig. S4, Supplementary Material online) (Rodrigues et al. 2016). In the bacterial cell, several other parameters such as DHFR abundance, catalytic efficiency (*k*_cat_/*K*_m_), thermal stability, availability of nutrients and metabolites, accumulation of excess DHF, and the need for THF can contribute to bacterial fitness in the presence of TMP. Finally, we engineered mutant *E. coli* strains by replacing WT *folA* gene with its variants carrying single mutations. All of the engineered *E. coli* strains with single DHFR mutations were viable (fig. 2*G*) and had elevated TMP resistance compared with their parental MG1655 strain (fig. 2*H*).

In summary, all DHFR mutations except the L28R and M20I mutations decreased both substrate and inhibitor binding with the exception of M20I which did not have a significantly different *K*_i_ value compared with the WT DHFR. On the other hand, the L28R mutation increased substrate affinity and decreased catalytic rate suggesting the existence of newly formed interactions between the mutated DHFR protein and its substrate (DHF). The catalytic efficiency of other DHFR mutants exhibited decreasing or increasing phenotypes. We conclude that the resistance-conferring mutations in DHFR are phenotypically diverse suggesting the presence of distinct resistance mechanisms.

### TMP-Free Enzymatic Velocities of DHFR Mutants Correlate Well with TMP-Free Growth Rates of *E. coli* Mutants Carrying Corresponding DHFR Mutations

Resistance-conferring mutations are rarely found in bacteria isolated from pristine environments and this observation is generally attributed to the fitness costs of resistance-conferring mutations. In the case of enzymes such as DHFR, where multiple resistance-conferring mutations are sequentially fixed, it is not clear how that many mutations can be tolerated while sufficient enzymatic activity is maintained for organismal survival. To address this question, we selected six of the mutations listed in figure 2*F* (P21L, A26T, L28R, W30G, W30R, and I94L) and synthesized a DHFR mutant library where we had all 48 (3^1^ × 2^4^) possible combinations of these mutations. We selected these six mutations because they had diverse effects on the catalytic efficiency of DHFR. Fortunately, we had access to a previously created library of *E. coli* mutants with all combinations of the listed DHFR mutations (Palmer et al. 2015). We purified and characterized all of the mutant DHFR enzymes as previously described (*k*_cat_, *K*_m_, and *K*_i_ values listed in supplementary table S4, Supplementary Material online). Next, we measured growth rates of the *E. coli* mutant library (supplementary fig. S5, Supplementary Material online) that carry the same DHFR mutations in various conditions (different temperature, different glucose concentrations, and different casamino acids concentrations) (supplementary fig. S5, Supplementary Material online). We found that enzymatic activity of DHFR mutants in the absence of TMP (*V*_0_, eq. 1), calculated at saturating [DHF], correlated well with the TMP-free growth rates of *E. coli* mutants with corresponding DHFR mutations (*r *=* *0.46–0.58, *P *<* *10^−3^, Pearson correlation test). The correlations between growth rates and other biochemical parameters such as *k*_cat_ or *k*_cat_/*K*_m_ were less significant (for *k*_cat_: [*r *=* *0.33, *P *<* *10^−3^]; for *k*_cat_/*K*_m_: [*r *=* *0.06, *P *<* *10^−3^], Pearson correlation). We note that the 12.5-µM DHF concentration is in good agreement with the previously measured in vivo DHF concentrations in which both reduced and oxidized species of folate concentrations were in the range of ∼10 µM (Kwon et al. 2008). These experiments and the resulting analyses suggested that *V*_0_, the substrate reduction rate of DHFR in the absence of TMP, is a good predictor of bacterial fitness, particularly when limited nutrients are provided to bacterial populations and bacterial cells are grown in the absence of TMP.

### Effects of Mutations on the Catalytic Power of DHFR Were Largely Context Dependent Due To Epistasis between Mutations

**Fig. 3.**
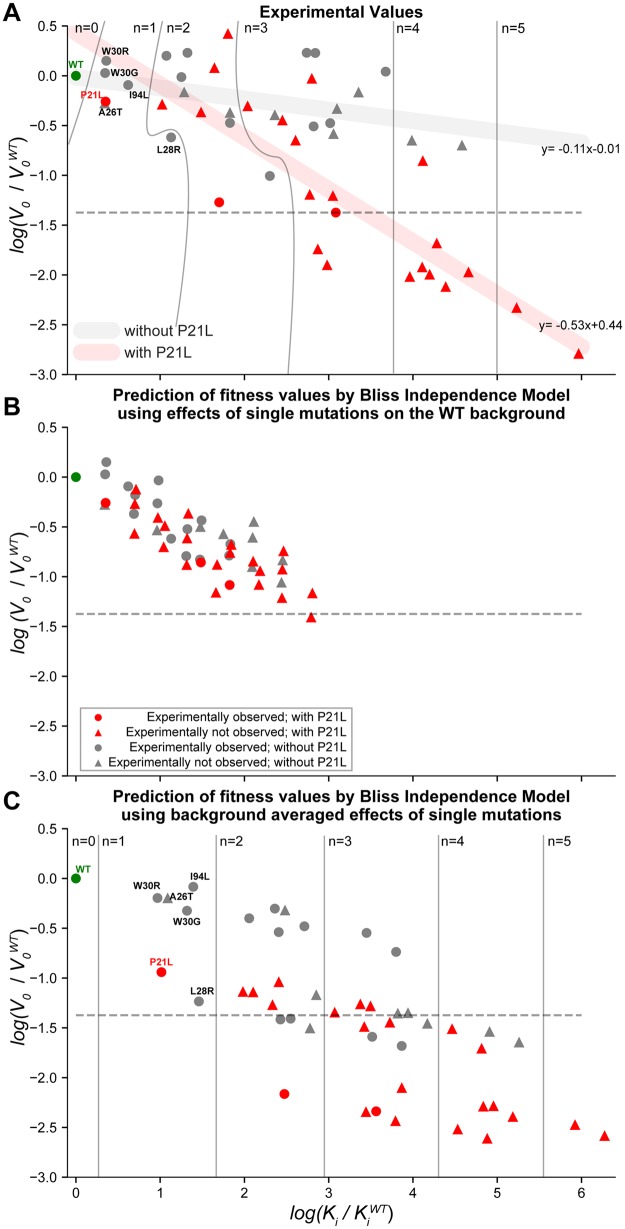
Combined effects of resistance-conferring mutations deviate from fitness values predicted by additivity model. (*A*) *V*_0_ versus *K*i values of the 48 DHFR mutants are plotted. Curved and straight lines are used to separate mutants with different number of mutations. Horizontal dashed line shows the minimum *V*_0_ value for a DHFR mutant that was observed in the morbidostat experiment. Red markers show mutants with P21L mutation. Gray markers show mutants without P21L mutation. Circle markers show mutants that are observed in evolution experiments. *V*_0_ values bifurcate depending on the presence of P21L mutation (red thick line). Same symbols are used in all three panels. (*B*) Predicted *V*_0_ and *K*i values for multiple DHFR mutants by an additivity model using the *V*_0_ and *K*i values measured for DHFR variant with single mutations (relative to the WT DHFR). These predictions significantly deviate from experimental observations (both for *V*_0_, and for *K*i [Student’s *t*-test, *P* < 10^−3^]). This model underpredicts *K*i values by a factor of 0.27 ± 0.35 and overpredicts *V*_0_ values by 3.34 ± 0.35 (mean ± standard deviation; supplementary fig. S8 and table S4, Supplementary Material online). (*C*) Predicted *V*_0_ and *K*i values for multiple DHFR mutants by an additivity model using the (geometric) mean effects of single mutations on all possible genetic backgrounds (supplementary table S5, Supplementary Material online). This model overpredicts *K*i values by a factor of 6 ± 3.96 and underpredicts *V*_0_ values by 0.35 ± 0.39 (mean ± standard deviation; supplementary fig. S8 and table S4, Supplementary Material online). The bifurcation observed in panel (*A*) disappears in both analyses summarized in panels (*B*) and (*C*).

In order to qualitatively understand the evolutionary trade-offs in DHFR evolution, we plotted *V*_0_ values against the corresponding *K*_i_ values for DHFR mutants. To our surprise, *V*_0_ values exhibited a bifurcation in this geometric representation (fig. 3*A*). DHFR mutants either had enzymatic activities comparable to their WT ancestor or significantly lost their enzymatic activities, some of which displaying almost no activity. All of the mutants that lost enzymatic activity carried the P21L mutation (fig. 3*A*, red triangles and circles). This bifurcation behavior could not be explained by any other single mutations (supplementary fig. S6, Supplementary Material online). In addition, none of the mutants that were detected in the morbidostat (fig. 3*A*, gray and red circles) had *V*_0_ values lower than 4% of the WT *V*_0_ (fig. 3*A*, horizontal dashed line). We note that almost all of the DHFR variants observed in the morbidostat appeared in the background of a promoter mutation that increases DHFR levels (fig. 1*B*, insert). Therefore, all the observed mutants in the morbidostat are predicted to have DHFR activity equivalent to 40–80% of the WT DHFR (*V*_0_).

Epistasis can simply be defined as the deviation from additivity when two or more genetic or environmental perturbations co-occur. In the absence of epistasis, the effects of genetic or environmental perturbations are independent and the effects of multiple mutations should simply add up to the sum of the individual effects of mutations (supplementary fig. S7, Supplementary Material online). In order to test the existence of epistatic interactions among DHFR mutations, we asked whether the *K*_i_ and *V*_0_ values deviated from the *K*_i_ and *V*_0_ values predicted by assuming additivity (no epistasis) (Bliss 1939). As shown in figure 3*B*, when the individual effects of six single mutations on the WT DHFR are used to calculate *K*_i_ and *V*_0_ values assuming additivity (Bliss 1939), the predicted *K*_i_ and *V*_0_ values significantly deviate from the experimentally measured ones (Student’s *t*-test, *P *<* *10^−3^). The predicted *V*_0_ values do not display a bifurcation and steadily decline as the number of DHFR mutations increase. Also, the predicted *K*_i_ values are not as large as the experimentally measured values (fig. 3*A* and *B* and supplementary fig. S8, Supplementary Material online). When we instead utilize the mean effects of single mutations on all possible genetic backgrounds in our mutant library (supplementary fig. S9 and table S5, Supplementary Material online), we can better estimate *K*_i_ values (fig. 3*C*). However, the bifurcation we observed in figure 3*A* still disappears and several of the mutants have lower predicted *V*_0_ values compared with the experimentally measured ones (supplementary fig. S8, Supplementary Material online). These observations clearly suggest the existence of epistasis among the six DHFR mutations we studied.

### High-Order Epistasis in Catalytic Power of DHFR (*k*_cat_ and *K*_m_) Creates a Rugged Fitness Landscape under TMP Selection

**Fig. 4.**
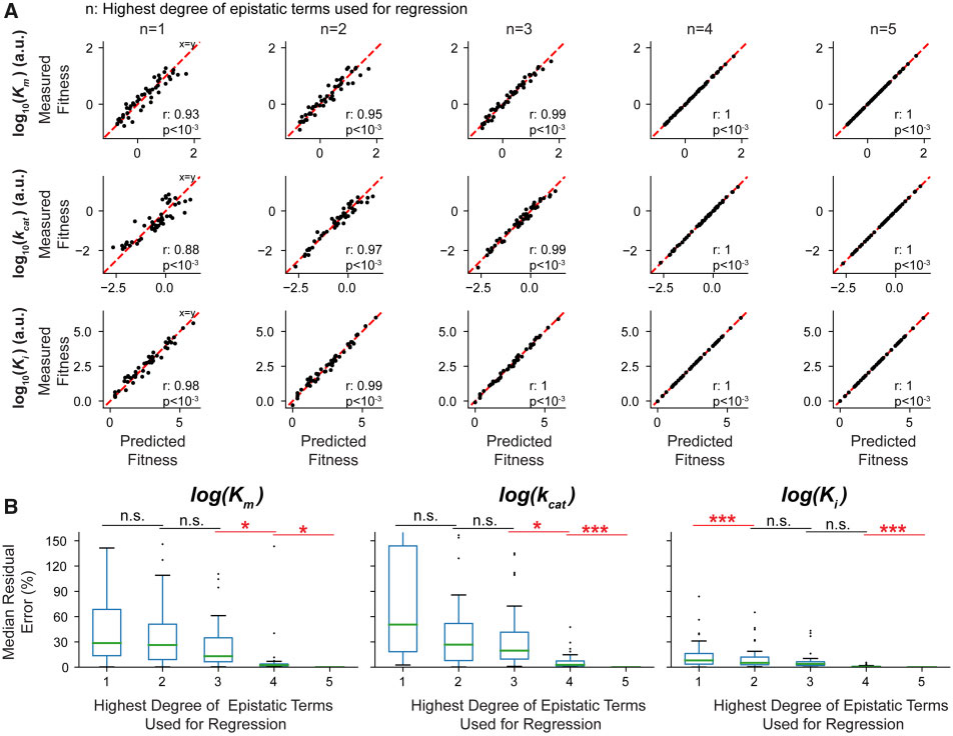
Epistasis between resistance-conferring DHFR mutations is high order for substrate binding and catalysis (*k*_cat_ and *K*_m_). (*A*) A linear regression model is used to predict fitness information stored in epistatic terms with increasing orders. Correlations between predicted fitness values of all genotypes using *n*th order epistatic terms and the measured fitness values are calculated. (*B*) Median residual errors for predicted fitness values as function of degree of epistatic terms used in regression. First order epistatic terms are sufficient to recover experimental *K*_i_ values with ∼10–20% residual error. However, at least second and third order epistatic terms are required to recover experimental *K*_m_ and *k*_cat_ values with ∼10–20% residual error.

We quantified epistatic interactions between the six DHFR mutations (P21L, A26T, L28R, W30G, W30R, and I94L) by utilizing a linear regression model (Materials and Methods) (Poelwijk et al. 2016). Briefly, we attempted to recover fitness values of all DHFR alleles using epistatic terms between mutations. In a biological system, if effects of mutations are independent, there is no epistasis and hence one can predict the fitness of genotypes with multiple mutations by simply adding the effects of single mutations. As shown in figure 4*A*, using linear regression, we were able to effectively predict *K*_i_ values for all DHFR mutants with up to five mutations by using only the first order epistasis terms (yielding ∼10–20% residual error). The extra information we gain from using higher order epistatic terms was relatively small (fig. 4*B*) indicating that measuring the mean effects of single mutations on *K*_i_ values will suffice to predict *K*_i_ values of DHFR mutants with any combination of the six DHFR mutations we studied. This analysis is consistent with our findings summarized in figure 3. However, for both *k*_cat_ and *K*_m_ values, to obtain a prediction power comparable with what we accomplished for *K*_i_, we needed to use at least up to third order epistatic terms and yet there was a big variance in the prediction performance (fig. 4*B*). This suggested that the effects of the mutations on DHFR’s catalytic activity were highly context dependent (supplementary fig. S7, Supplementary Material online) which make fitness landscape of DHFR rugged (Palmer et al. 2015). In other words, even knowing all of the pairwise interactions between these six mutations was not sufficient to predict fitness of DHFR variants carrying three or more mutations. Since DHFR fitness in TMP containing environment is a convoluted function of all *k*_cat_, *K*_m_, and *K*_i_ values, evolution of TMP resistance in the adaptive landscape becomes mostly unpredictable mainly because of high-order epistatic interactions in catalytic power of DHFR (*k*_cat_ and *K*_m_).

### Promoter Mutations Compensate Detrimental Effects of DHFR Mutations and Largely Increase Number of Plausible Evolutionary Trajectories

**Fig. 5.**
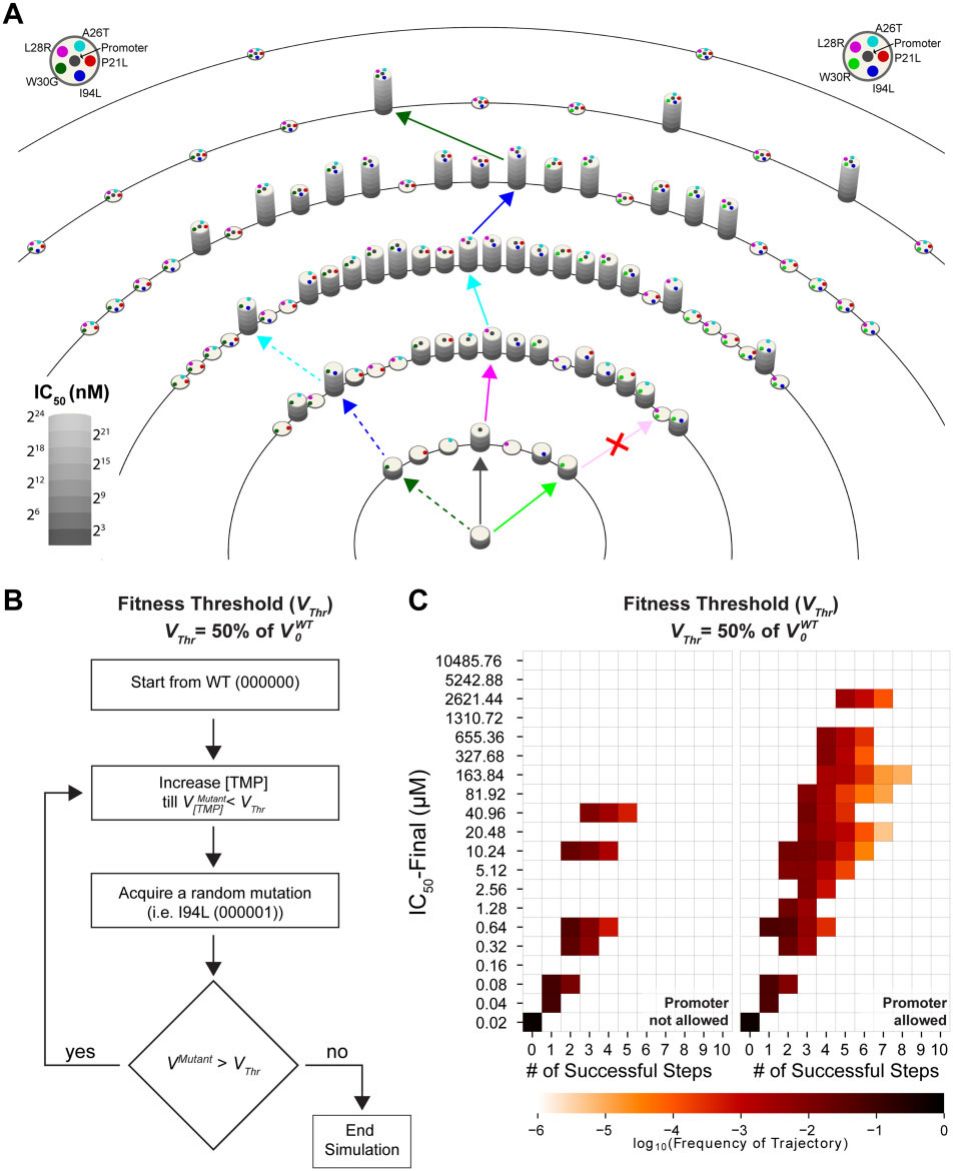
Simulated evolutionary trajectories leading to TMP resistance. (*A*) DHFR alleles are represented as cylindrical pillars. Atop of pillars, colored filled circles are used to show DHFR mutation. Heights of the cylinders correspond to TMP concentrations required to reduce the activity of mutant DHFR enzymes down to half of the *V*_0_ for the WT DHFR (*V*_0_^WT^). Note that several pillars have zero height because their activities never exceed half of *V*_0_^WT^ even in the absence of TMP. The trajectory represented with solid arrowed lines is one of the shortest and most common pathways leading to global maximum of the adaptive landscape. The trajectory represented with dashed arrowed lines lead to a local maximum of the adaptive landscape if the promoter mutation is not allowed. (*B*) Schematics summarizing the algorithm used in simulations. (*C*) Simulations analysis summarized in heat maps. In simulations where the promoter mutation is not allowed (left), trajectories are shorter compared with the trajectories where the promoter mutation is allowed (right). If the promoter mutation is allowed, an increased number of trajectories lead to adaptive peaks with higher TMP resistance levels.

Evolution of TMP resistance is a random search for mutational trajectories that lead to the resistant DHFR genotypes without sacrificing catalytic activity. We ran computer simulations to visualize and quantify plausible evolutionary trajectories leading to TMP resistance. As demonstrated in figure 5, for every DHFR allele, we calculated DHFR activity (*V*) as a function of TMP concentration. In figure 5*A*, as a measure for fitness, we use TMP concentrations necessary to reduce mutated DHFR enzymes’ activities down to 50% of *V*_0_ for the WT DHFR (*V*_0_^WT^). In this panel, DHFR mutants are represented as cylindrical pillars with heights proportional to TMP concentrations necessary to reduce mutated DHFR enzymes’ activities down to 50% of *V*_0_ for the WT DHFR (*V*_0_^WT^). Colored filled circles on the upper surface of the cylinder represent DHFR mutations. We note that this landscape dynamically changes (supplementary video 1, Supplementary Material online) as we increase TMP concentrations used in our calculations. In these calculations (eq. 1), we used a saturating DHF concentration (12.5 µM) which is in the physiological range and we assumed a 10-fold increase in DHFR expression due to the promoter mutation (fig. 1*B*, insert). Alleles are grouped according to the number of mutations they have. We then ran stochastic simulations where we allow DHFR to acquire mutations as TMP dosage is gradually increased (fig. 5*B*). All simulations start from the WT DHFR allele and the activities of all DHFR alleles are calculated at every TMP concentration. In these simulations, we assume that any DHFR mutant that has activity (*V*) less than half of the WT DHFR activity (*V*_0_^WT^, no TMP) goes extinct unless they acquire a beneficial mutation. In our simulations, we allow DHFR to obtain or lose one of the seven mutations (promoter, P21L, A26T, L28R, W30G, W30R, and I94L) if activity of the mutant is about to drop below half of *V*_0_^WT^. Any of these mutations can be added, converted (W30R → W30G, W30G →W30R) or reverted (e.g., L21 mutant to P21). As shown in figure 5, we observed several genetic trajectories that arrive at local or global maxima. We repeated these simulations 10^6^ times and quantified relative abundance of mutational trajectories (fig. 5 and supplementary table S6, Supplementary Material online).

Mutational trajectories that lead to high TMP resistance peaks typically accumulated up to five mutations and the majority of these trajectories reached to the fitness peaks in five to seven genetic steps. Several viable trajectories included more than five mutational steps mainly because reverting the P21L mutation back to WT (L21P) significantly improved DHFR fitness in many genetic backgrounds. We then ranked all of the genetic trajectories that reach to high TMP resistance by taking the least possible number of steps and calculated the likelihood of each mutation in the adaptive landscape (supplementary table S6, Supplementary Material online). We have repeated these simulations using lower fitness thresholds (i.e., 1% of *V*_0_ for the WT DHFR) and showed that number and length of evolutionary trajectories that reach to fitness peaks drastically increase if minimum fitness thresholds are assumed to be lower (supplementary fig. S10, Supplementary Material online).

We computationally tested the effect of promoter mutations in DHFR evolution (fig. 5*C*). To do this, we ran simulations where all of the DHFR alleles with promoter mutations were eliminated and we compared these simulations with those that allow the promoter mutation. We found that number of plausible mutational trajectories that lead to TMP-resistant genotypes significantly diminishes if the promoter mutation is not allowed (fig. 5*C*). When promoter is not allowed, only ∼1.29% of the simulated trajectories reach to genotypes that survived in 32-µM TMP which is considered as resistant in clinical settings. There are only 60 unique trajectories which acquired one or more DHFR mutations and increased TMP resistance. However, when promoter mutation is allowed, ∼5.59% of the simulated trajectories reach to genotypes that survived in TMP concentrations between 32 µM and ∼2.58 mM. In this case, 2,581 unique trajectories acquired one or more DHFR mutations and increased TMP resistance (supplementary table S6, Supplementary Material online). This reduction effect is mainly due to elimination of half of the possible genetic combinations between the six resistance-conferring mutations we studied and also elimination of the compensatory effect of the promoter mutation. Thus, number and length of plausible evolutionary trajectories, as well as the maximum possible TMP resistance significantly diminish in the absence of the promoter mutation. Therefore, in the absence of promoter mutation, DHFR evolution becomes more predictable.

We conclude that the first plausible mutation in DHFR evolution is expected to be one of the promoter, W30R, or W30G mutations. Indeed, the c-35t and W30R mutations were previously found in clinically isolated *E. coli* strains (Flensburg and Skold 1987). Once the promoter mutation is fixed, *E. coli* cells will accumulate a mutation in the coding region of the *folA* gene. This mutation can be any of the mutations we observed in our morbidostat experiments (fig. 1*D* and supplementary table S7, Supplementary Material online) since there will be no epistatic interactions at this point and the promoter mutation makes the adaptive landscape of DHFR less predictable by compensating for diminished catalytic activities of resistance-conferring DHFR mutation(s) (fig. 5*C*). After the first mutation in the coding region is fixed, acquisition of further mutations will largely be dictated by the epistatic interactions. Mutations that have synergistic or additive epistatic interactions (i.e., L28R) with other DHFR mutations are more likely to get fixed in the evolving populations, whereas mutants carrying mutations that antagonize other DHFR mutations (i.e., P21L) will most likely be outcompeted due to their poor catalytic efficiencies.

### Structural Evaluation of DHFR with Single Mutations Reveals Distinct Resistance Mechanisms at the Molecular Level

We utilized MD simulations to study the structural changes associated with the experimentally observed TMP resistance-conferring mutations in DHFR. DHFR is formed of eight stranded β-sheets and four contiguous α-helices (Sawaya and Kraut 1997; Dams et al. 2000; Heaslet et al. 2009). The enzyme is divided by the active site cleft into two subdomains: the adenosine binding subdomain and the major subdomain. The former (residues 38–88) provides the binding site for the adenosine moiety of the cofactor (NADPH). The latter subdomain consists of ∼100 residues and contains three loops on the ligand binding face that surrounds the active site. Of particular interest is the M20 loop located directly over the active site, protecting it from the solvent, and involved in the regulation of the hydride transfer step (Sawaya and Kraut 1997). It is found in one of three conformations, known as the open, occluded, or closed states (Sawaya and Kraut 1997; Miller et al. 2001). In our computer simulations, we have used the closed state as the starting structure (PDB ID: 1rx2) (Sawaya and Kraut 1997). For each mutant studied as well as the WT DHFR, we have compiled MD trajectories for both the DHFR/NADPH/DHF (fig. 2*A*, green) and the DHFR/NADPH/TMP complexes (Materials and Methods) (Abdizadeh et al. 2017).

**Fig. 6.**
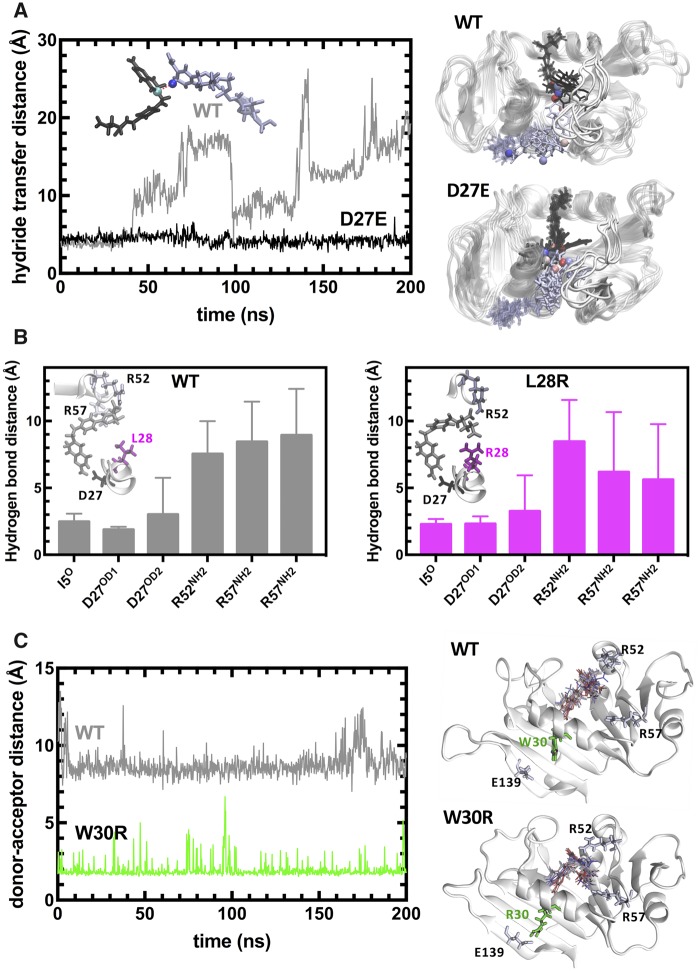
Molecular mechanisms operating in the DHF bound dynamics of DHFR for the three frequently observed DHFR mutations. (*A*) D27E replacement alters hydride transfer distance between the cofactor (NADPH) and the substrate (DHF). The measured distance is between the cyan and blue spheres shown in the inset for the crystal structure positioning of NADPH (black) and DHF, which is readily lost in the WT structure as in all the other simulations of the single mutants except for D27E. Dynamical motions of NADPH and DHF are displayed on the right. (*B*) L28R mutations yields extra direct hydrogen bonds with DHF and stabilizes it in the binding pocket. The distance between the donors and acceptors of the hydrogen bonds originally present in the crystal structure is monitored throughout the MD trajectories with their averages and standard deviations displayed. Although the original hydrogen bonds are lost in both the wild type and the L28R mutant, there are many new hydrogen bond donor sites on the R28 side chain, maintaining a dynamical hydrogen bonding ecology around the substrate. (*C*) W30R mutation releases the tension in the tight binding pocket by forming a salt bridge with E139. The distance between the E139 acceptor (O- group) and the closest heavy atom of residue 30 is plotted for the wild type and the W30R mutant. In the latter case a salt bridge is established between the side groups frequently, relaxing the tight binding pocket where the substrate resides. As shown on the right, DHF maintains a position between the stabilizing R52 and R57 side chains in the mutant while the contacts with R57 group is lost in the wild type.

We have monitored the WT and all 11 single mutant sets of MD trajectories corresponding to those listed in figure 2*F* to decipher the molecular mechanisms that lead to TMP resistance. We note that although these mutations are observed with various frequencies in the morbidostat trajectories as displayed in figure 1*D*, only nine of them appeared as the first coding region mutation in *folA* (supplementary table S7, Supplementary Material online). Amongst these, D27E, L28R, and W30R are three most frequently observed mutations in the morbidostat and interestingly, these are also the only cases where significant structural changes were identified in the dynamical trajectories (fig. 6).

Amongst the WT and all single mutants we analyzed, the D27E mutant is the only one where the hydride transfer distance is kept at an optimal precatalytic range (fig. 6*A*). We note that in all mutations we studied, the M20 loop never leaves the closed conformation in favor of the occluded form which triggers the reduction of DHF into THF. Nevertheless, the longer side chain (glutamic acid) of the D27E mutant renders the moiety more flexible and dynamically maintains the ligand at an optimal distance, keeping it ready for the hydride transfer once this rare event takes place, hence explaining the increase in *k*_cat_ for the D27E mutant (fig. 2*F*). On the other hand, the L28R mutation leads to the formation of extra hydrogen bonds between the enzyme and DHF, thus stabilizing its conformation (Abdizadeh et al. 2017). In figure 6*B*, we display the average distance of hydrogen bonds formed between the enzyme and DHF. Although the pterin ring of DHF is permanently engaged in the binding pocket (as evidenced by the hydrogen bond distances to I5 and D27), the *p*-aminobenzoyl glutamate tail is mobile in WT DHFR. In contrast, this mobility is significantly reduced in the L28R mutant due to the extra interactions with the side chain. Unlike D27E and L28R, the effect of W30R on the dynamics of DHF is indirect. In this case, the R30 side chain of the mutant forms a salt bridge with the side chain of E139 residing on the β sheet supporting the catalytic region (fig. 6*C*). The distance between the two residues is reduced from a baseline value of ∼8 Å to ∼2 Å. This interaction slightly opens the tight binding pocket so that the DHF *p*-aminobenzoyl glutamate tail motions are accommodated in the region between R52 and R57 residues, whereas the glutamate tail is more disordered and closer to R52 residue in the WT DHFR. Reduced interactions between the *p*-aminobenzoyl glutamate tail and the enzyme leads to weaker substrate binding and higher catalytic rate. In the rest of the DHF-bound MD simulations of the single mutants, the changes in the dynamics of the system are subtle.

### MD Simulations Demonstrate the Context-Dependent Effects of DHFR Mutations at the Atomic Level

Epistatic interactions in biological systems are common and were previously reported by several researchers. However, in most cases, the structural basis of epistasis was not sufficiently explained (Weinreich et al. 2006; Toprak et al. 2011). To study structural basis of epistasis between resistance-conferring DHFR mutations, we utilized MD simulations for a subset of DHFR alleles including all combinations of the mutations A26T, L28R, and I94L. In addition, we traced the effect of adding P21L mutation to some of these mutants to trace how this mutant drastically reduces enzymatic activity (fig. 3). Among these, L28R is frequently observed as the first coding region mutation in the morbidostat, whereas A26T and I94L are observed later in evolution experiments (supplementary table S7, Supplementary Material online).

**Fig. 7.**
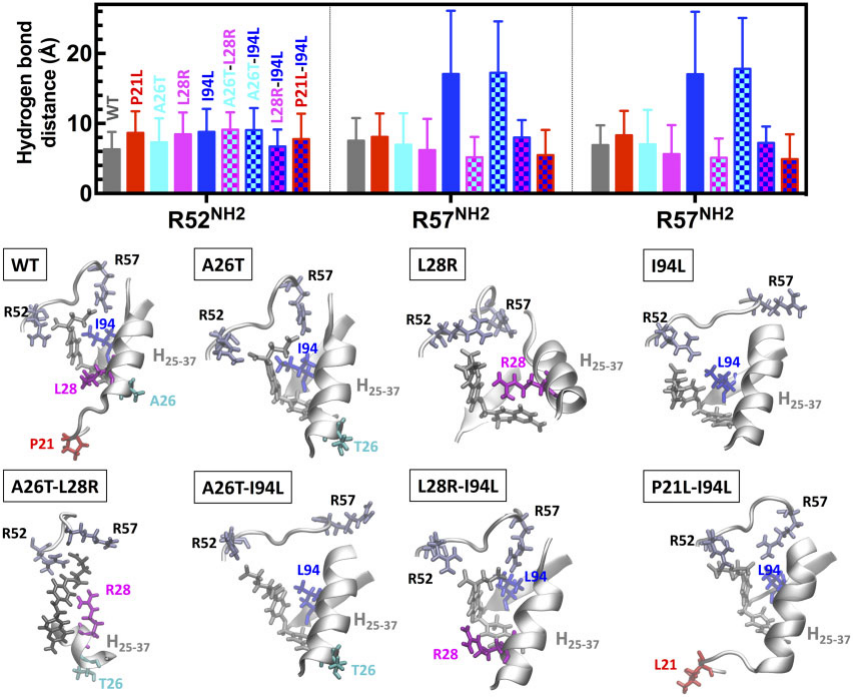
Epistasis between resistance-conferring DHFR mutations are largely due to interactions of the mutated enzyme with the *p*-aminobenzoyl glutamate tail of DHF. (top) Selected hydrogen bond distances between *p*-aminobenzoyl glutamate tail of DHF and DHFR for WT and a series of single and double mutants. (bottom) Representative binding pocket poses. Helix spanning residues 25–37 (H_25–37_), partial loop spanning residues 52–57 and the ligand (gray stick) is displayed in all figures. A26T is neutral, L28R stabilizes DHF by mechanism described in figure 6*B*. I94L mutation exacerbates substrate binding of DHFR by altering tight interactions with the *p*-aminobenzoyl glutamate tail of DHF in the binding pocket, allowing the R57 side chain to flip out. L28R mutation is a highly epistatic mutation; together with either A26T or I94L, the L28R further stabilizes the substrate in the pocket. P21L-I94L double mutation also rescues the negative effect of I94L, whereas A26T-I94L does not.

We demonstrate the context dependence of the observed dynamics by focusing on four specific examples involving double mutations. We traced the signature hydrogen bonds (H bonds between substrate and the side chains of I5, D27, R52, and R57) between the enzyme and the substrate (fig. 7) and found that hydrogen bonds between the I5 and D27 side chains in the studied mutants were always close to their native values in the WT DHFR. However, those between the R52 and R57 side chains and DHF showed significant variations (displayed in figure 7, averaged over the last 100 ns of the trajectories.) For the single mutants, we do not find any significant dynamical changes in the MD trajectories for P21L and A26T mutations. We note that the common reduction in the *k*_cat_ value due to the P21L mutation (fig. 2*F*) possibly occurs on time scales slower than the sub-microsecond observation window of our MD simulations; for example, due to the modified dynamics of the catalytic M20 loop, whose conformational switch occurs on the time scale of ∼2–40 s^−1^(Schnell et al. 2004). Meanwhile, the I94L mutant completely loses interactions with the R57 side chain since the slight change in the isomerization of the side chain leads to more prolonged interactions with the aromatic ring of DHF, distorting the tight binding pocket. As a result, the R57 side chain flips out of the pocket to the other side of the helix spanning residues 25–37 (fig. 7).

L28R mutation leads to the formation of extra hydrogen bonds with DHF. We found that together with A26T, this effect becomes even stronger, fixing the position of DHF to the space between R52 and R57 residues (fig. 7). Thus, although the A26T mutation alone causes subtle structural changes in our MD simulations, together with L28R, it benefits from a synergistic effect on DHF binding, with the polar side chain further stabilizing the network of hydrogen bonds in the pocket. The L28R mutation has a similar synergistic effect on the I94L mutation. Despite the tendency of the I94L mutant to interact strongly with the aromatic part of DHF, the binding pocket is not as easy to distort due to the presence of R28 interactions with the substrate, further stabilizing DHF. As expected by the outlined mechanism of action, addition of A26T to the I94L mutation does not lead to the same synergistic effect. Interestingly, although P21L mutation mostly impairs catalytic activity of DHFR, the P21L mutation rescues I94L mutant. In this case, the more flexible L21 allows distortions of the tight binding pocket without letting the R57 side chain to flip out (fig. 7). We note that these mutations significantly decrease the binding propensity of the inhibitor, as measured by the *K*_i_ values listed in supplementary table S3, Supplementary Material online. DHF escapes this fate due to the extra interactions of the larger ligand with the side chains of the enzyme. Running longer MD simulations for all possible combinations of DHFR mutations was beyond our computational capacity but even the analysis of this small subset of DHFR mutants demonstrated the context dependent effects of DHFR mutations at the molecular level.

## Discussion

DHFR is a ubiquitous enzyme commonly used as a drug target in antibacterial, anticancer, and antimalarial therapies (Schnell et al. 2004). Developing a better understanding of the evolution of drug resistance through sequential accumulation of DHFR mutations is therefore an important scientific task to help improve drug therapies. Our experimental findings and computational analyses demonstrate that DHFR is a highly evolvable enzyme that can maintain its catalytic activity while accumulating multiple resistance-conferring mutations. Experimental and computational analyses of six of these mutations demonstrate the prevalence of epistatic interactions between them which imply the ruggedness of the adaptive landscape (fig. 5) that lead to TMP resistance.

Epistasis between resistance-conferring mutations in *E. coli* DHFR and pfDHFR was previously reported and quantified by engineering all possible combinations of a small number of resistance-conferring mutations (Lozovsky et al. 2009; Palmer et al. 2015). A similar analysis was also done for a beta-lactamase gene in the landmark study of Weinreich et al. (2006). These studies mainly utilized bacterial growth assays to quantify fitness effects of mutations and assessed the predictability for evolution of resistance. In another landmark study by Lunzer et al., where effects of amino acid changes in isopropylmalate dehydrogenase’s coenzyme choice were systematically studied, it was demonstrated that each amino acid replacement additively contributed to the function of isopropylmalate dehydrogenase’s enzymatic function, and that the epistasis comes from nonlinear mappings from enzymatic phenotypes to fitness (Lunzer et al. 2005). In this study, by utilizing both biochemical assays and growth rate measurements, we deconvolved epistasis between resistance-conferring mutations and demonstrated that epistasis was largely due to changes in catalytic activity of the mutant DHFR enzymes. We also showed that epistatic interactions and the compensatory effects of promoter mutations significantly diminish our ability to predict DHFR evolution in the presence of TMP-induced selection.

In a recent study, Rodrigues et al. investigated epistasis between three of the mutations we studied (P21L, L28R, and W30R) and developed an elegant framework to predict fitness of *E. coli* strains carrying DHFR alleles with combinations of these three mutations by using biophysical properties of DHFR mutations (Rodrigues et al. 2016). However, because of the small number of possible combinations (2^3^) of DHFR mutations they studied, they were not able to detect the P21L-caused bifurcation in the fitness landscape we report here (fig. 3). For a larger set of combinations of DHFR mutations that include the P21L, we found that fitness prediction of DHFR alleles is more challenging. Using the available biochemical fitness values we have, we were able to identify partial correlation between catalytic power and bacterial growth rates of DHFR mutants (supplementary fig. S5, Supplementary Material online). However, we were not able to demonstrate a direct correlation between TMP resistance and biochemical parameters we measured (supplementary fig. S4, Supplementary Material online). We note that predicting TMP resistance levels might be possible by using extra biophysical parameters such as thermal stability and abundance of DHFR mutants as was demonstrated by Rodrigues et al. (2016).

Our analysis suggests that although predicting DHFR evolution is a difficult task, it might still be possible to steer evolution of TMP resistance towards clinically less challenging genotypes. Among all the mutations we studied, promoter and L28R mutations can potentially be targeted to reduce the number of plausible evolutionary trajectories and TMP resistance. If practical limitations are addressed, specifically targeting the promoter mutation by utilizing one of the novel gene editing tools or sequence-specific morpholino oligomers will substantially decrease both the number of accessible trajectories and maximum resistance levels (fig. 5 and supplementary fig. S10, Supplementary Material online) (Jiang et al. 2013; Ayhan et al. 2016). Also, since the L28R mutation has a distinct molecular mechanism that increases its relative preference for the substrate over the drug molecules (fig. 6), it might be possible to design L28R-specific DHFR inhibitors that will mimic DHF without losing its specificity against bacterial DHFR. Since L28R mutation is observed in almost 80% of all morbidostat trajectories and is synergistically interacting with several mutations, an L28R-specific inhibitor will substantially impede evolution of TMP resistance.

## Materials and Methods

### Growth Rate Measurements

All *DHFR* mutant strains were constructed in MG1655 attTn7::pRNA1-tdCherry (*NDL47*) (gift from Johan Paulsson, HMS). Detailed procedures for making mutant strains can be found in reference (Palmer et al. 2015). Bacterial cultures were grown at 30 °C in M9 minimal medium supplemented with 0.4% glucose (Fisher Scientific B152-1), 0.2% amicase (MP Biomedicals 104778), 2-mM MgSO_4_ (Fisher Scientific M63-500), and 100 µM of CaCl_2_ (Fisher Scientific S25222A). Overnight grown cultures normalized to optical density (OD) of 0.001. Plates were incubated in 30 °C with continuous shaking in Liconic Shaking Incubator and growth is measured with Tecan Plate Reader Infinite M200. Background optical density levels (OD ∼ 0.04) are substracted from all wells. Growth rates are calculated by making an exponential fit to growth curve when bacterial growth is in its exponential phase.

### Intracellular DHFR Protein Abundance Measurements

*Escherichia coli NDL47* cells were grown overnight, and final OD600 was adjusted to unity. These cells were then diluted by 10^4^-fold in 5 ml of M9 minimal media (supplemented with 0.4% glucose and 0.2% amicase) and grown for 6 h at 37 °C (220 rpm). Cells were then washed three times with cold PBS buffer (pH 7.4), and bacterial pellets were lysed in 1× Laemmli sample buffer (5 ml/OD). Equivalent amounts of the cell lysates (10 μl of the above sample) from each set were electrophoresed in a 4–15% precast polyacrylamide gel (561081; BIO-RAD), and western blotting was performed following standard procedures. DHFR antibodies are kindly provided to Kimberly Reynolds by Shimon Bershtein. IR-labeled secondary antibodies (IRDye 800CW [926–32213] and IRDye 680RD [925–68072]; Li-COR) were used for detection. DHFR protein amount was quantified using an ODYSSEY infrared imaging system (LI-COR).

### Steady-State Kinetic Measurements

Reactants of DHFR reaction (DHF [Sigma-Aldrich D7006] and NADPH [Sigma-Aldrich N7505]) has absorbance at 340 nm which the products (THF and NADP^+^) do not absorb light. Concentrations of DHF and NADPH have been measured using molar concentration coefficients of 6,200 M^−1^ cm^−1^ at 340 nm and 28,000 M^−1^ cm^−1^ at 282 nm (Fierke et al. 1987). Using LAMBDA 650 UV/Vis Spectrophotometer, we measured reaction progression with 1-s resolution with two cells. First cuvette is sample cuvette containing the reaction components (DHFR, DHF, and NADPH) and the second is reference cell contains only NADPH and DHFR in it. Biochemical measurements were done at 25 °C in MTEN buffer (pH ∼ 7) which includes, 50-mM MES hydrate (Sigma-Aldrich M8250), 25-mM Tris–Base (Fisher Scientific B152-1), 25-mM ethanolamine hydrochloride (Sigma-Aldrich E6133), 100-mM NaCl (Fisher Scientific S271-3), and 5-mM DTT (Fisher Scientific BP172-25) which is added fresh before starting the reaction. MTEN solution containing DHFR protein and 200-µM NADPH is prepared and 12.5-µM DHF and 200-µM NADPH solution is added preceding the data collection. Data collection is stopped when all the DHF is consumed which happens when the curve reach a plateau down below zero. Data analysis is done as explained in the main text (fig. 2*B* and *C* and supplementary fig. S2, Supplementary Material online). Data analysis software can be found in https://github.com/ytalhatamer/EnzymeKinetics-Matlab; last accessed April 16, 2019.

### Calculating Inhibition Constant (*K*_i_) for TMP

To calculate inhibition constants for TMP, we used initial rates of the reactions with saturating concentrations of DHF and NADPH with different TMP concentrations. We fit equation (1) to predict *K*_i_ values as demonstrated in figure 2*D* and *E*.

### Protein Overexpression and Purification

All combinations of six mutations of *folA* gene at five sites (I94L, W30R, W30G, L28R, A26T, and P21L) are constructed by using Quick-Change Site-Directed Mutagenesis kit (Stratagene). 6XHis Tag is added on C-terminal of the protein sequence. Constructs are cloned into the expression plasmids (pET24a-KanR) for further protein purification. BL21 cells are transformed with pET24a-*folA*-6xHisTag were grown overnight in selective media (LB + Kan) and then diluted 100 times into TB media for further growth at 30 °C. Protein overexpression induced when OD reached 0.6–0.8 using 250-µM IPTG at 18 °C with 220-rpm shaking. Recombinant proteins are further purified using Ni-NTA columns (Qiagen) and dialyzed overnight using dialysis buffer containing 50-mM Tris–Base, pH 8.0, 0.5-M NaCl, and 400-mM imidazole (Sigma Aldrich 792527).

### Epistasis Calculations and Linear Regression Model

A linear regression model is used to predict fitness of DHFR alleles by using epistatic interactions terms between DHFR mutations using the following equation:

Y=X· β+ϵ.

Here, *Y* stands for phenotypes, X stands for regression matrix, β stands for regression coefficients, and ϵ stands for residual noise terms. X matrix is used to determine which regression coefficients will be used for a specific genotype and it can be recursively created as following:

$X_{n+1}=\left[\begin{array}{cc} X_{n} & 0\\ X_{n} & X_{n} \end{array}\right]\;(n=0:\;6)\;$where $X_{1}\;\mathrm{and}\;X_{2}$ are defined as below.

$${X_{0}=1\;[(\mathrm{only}\;\mathrm{Wild}\;\mathrm{Type}\;\left(\mathrm{WT}\right)]);\;X}_{1}=\left[\begin{array}{cc} 1 & 0\\ 1 & 1 \end{array}\right]\;\left(\mathrm{for}\;\mathrm{WT},\;\mathrm{and}\;I94L\right),$$

$$X_{2}=\left[\begin{array}{cccccc} 1 & 0 & 0 & 0 & 0 & 0\\ 1 & 1 & 0 & 0 & 0 & 0\\ 1 & 0 & 1 & 0 & 0 & 0\\ 1 & 1 & 1 & 1 & 0 & 0\\ 1 & 0 & 0 & 0 & 1 & 0\\ 1 & 1 & 0 & 0 & 1 & 1 \end{array}\right]\left(\mathrm{for}\;\mathrm{WT},\;I94L,\;W30R,\;\mathrm{and}\;W30G;W30R\;\mathrm{and}\;W30G\;\mathrm{cannot}\;\mathrm{coexist}\right).$$

The theory and algorithm is described in detail by Poelwijk et al. (2016).

### MD Simulations

The NAMD package is used to model the dynamics of the protein–water systems (Phillips et al. 2005). Solvation is achieved via the VMD 1.9.1 program solvate plug-in version 1.2 (Humphrey et al. 1996). The protein is soaked in a cubic solvent box such that there is at least a 10-Å layer of solvent in each direction from any atom of the protein to the edge of the box. The system is neutralized and 150 mM of ionic strength in all the simulations is maintained by adding a suitable number of K^+^ and Cl^−^ ions. The CharmM22 all-atom force field is used to model the protein and the TIP3P potential for water (Brooks et al. 1983; Abdizadeh et al. 2017). We have adopted the force field parameters for five-protonated DHF and TMP in two protonation states as reported in the literature (Garcia-Viloca et al. 2003). Periodic boundary conditions are imposed on the simulation boxes that have 60 × 67 × 58-Å^3^ dimensions. Long range electrostatic interactions are calculated by the particle mesh Ewald method (Darden et al. 1999) with a cutoff distance of 12 Å and a switching function at 10 Å. The RATTLE algorithm (Andersen 1983) is applied and a time step size of 2 fs in the Verlet algorithm is used. Temperature control is carried out by Langevin dynamics with a dampening coefficient of 5 ps^−1^. Pressure control is attained by a Langevin piston. All systems are first subjected to 10,000 steps of energy minimization with the conjugate gradients algorithm. The resulting structures are then run in the NPT ensemble at 1 atm and 310 K until volumetric fluctuations are stabilized and the desired average pressure is maintained.

MD simulation of the ternary complex of the DHF bound systems are constructed based on the crystallographic structure with PDB code 1rx2 (Sawaya and Kraut 1997). DHFR is complexed with folate and oxidized NADP (NADP^+^) in this native form. We protonate NADP and folate so that the former is in the reduced form (NADPH) and the latter is five-protonated DHF to model the stable state prior to the hydride transfer step.

In a separate set of MD simulations, we study the effect of TMP binding in its unprotonated (TMP) or ground state (TMP^+^) on the DHFR conformation. Since there are no crystal structures of *E. coli* DHFR with TMP, we have docked the inhibitor based on the coordination of equivalent residues of the TMP binding region of *Staphylococcus aureus* DHFR (PDB code: 2w9g) (Heaslet et al. 2009). Details of TMP binding site selection is provided in reference (Abdizadeh et al. 2017). For MD simulations of the various mutants of DHF, TMP, and TMP^+^ bound forms of DHFR, we mutated the WT structures in silico via BIOVIA Discovery Studio 4.0 package using build and edit protein tool (Dassault Systèmes BIOVIA 2015). For systems with multiple mutations, we substituted the native positions with the target mutations simultaneously. The solvation, ionization, minimization, and equilibration were performed as described for the WT systems. All MD simulations are 210-ns long, with the first 10 ns discarded for equilibration. Simulations for the WT cases were repeated to confirm the reproducibility of the results.

The mutants studied are as follows: The single mutants I5F, M20I, P21L, A26T, D27E, L28R, W30G, W30R, I94L, R98P, and F153S; all double mutant combinations of the A26T, L28R, and I94L sets; double mutant combinations of P21L with each of A26T, L28R, and I94L. Thus, we have carried out 210-ns-long simulations of 17 sets of mutants, with DHF, TMP, and TMP^+^ bound, leading to simulations of ∼8 μs in total, including the WT sets.

We use the approach in reference (Abdizadeh et al. 2017) to confirm the native form of TMP in the DHFR bound state, by monitoring the distribution of the native hydrogen bonds in the binding pockets. In all the sets, TMP^+^ remains tightly bound, whereas TMP flips in and out of the binding pocket throughout the simulation. We thus accept the protonated form of TMP to be the native form in all the systems; note that this is not the case for D27N and D27S mutants, as discussed at length in reference (Abdizadeh et al. 2017).

### Simulations of Protein Evolution and Visualization

Protein evolution simulations works on a DHFR mutational space (proteins as nodes and single mutation acquisition, conversion or reversion as lines). Simulation starts from WT in no TMP condition. TMP concentration gradually increases and at each drug concentration fitness landscape of DHFR mutational space is calculated. When drug concentration hits a value where enzyme activity is lower than threshold activity (simulations are separately carried out for 50%, 10%, 1%, 0.1% of WT enzyme activity at [TMP] = 0 nM) a random mutational step is taken (a mutation acquisition, conversion, or reversion). If the new mutant has lower activity than threshold, the simulation stops, otherwise the new mutation is fixed, and drug concentration starts increasing again till new mutants’ activity drops down to the threshold level (fig. 7*B*). Simulations are repeated for a million times to sample all possible unique trajectories. Visualization of the simulations is done by VPython, an open source software package for interactive 3D graphics (Scherer et al. 2000). Our script for producing figure 5*A*, and the supplementary video, Supplementary Material online, can be found in https://github.com/ytalhatamer/AdaptiveSeascape; last accessed April 16, 2019.

## Supplementary Material

Supplementary data are available at *Molecular Biology and Evolution* online.

## Supplementary Material

Supplement_Material_msz086
