# Supplementary material for: High-Order Epistasis in Catalytic Power of Dihydrofolate Reductase Gives Rise to a Rugged Fitness Landscape in the Presence of Trimethoprim Selection
Source: Mol Biol Evol. 2019 Apr 15;36(7):1533–50. doi: 10.1093/molbev/msz086 (PMC6573477; doi:10.1093/molbev/msz086)
Supplement: Supplement_Material_msz086 [file supplement_material_msz086.zip › Supplementary_Figures.pdf]

High-order epistasis in catalytic power of dihydrofolate reductase gives rise to a rugged fitness landscape in the presence of trimethoprim selection

Supplementary Figures

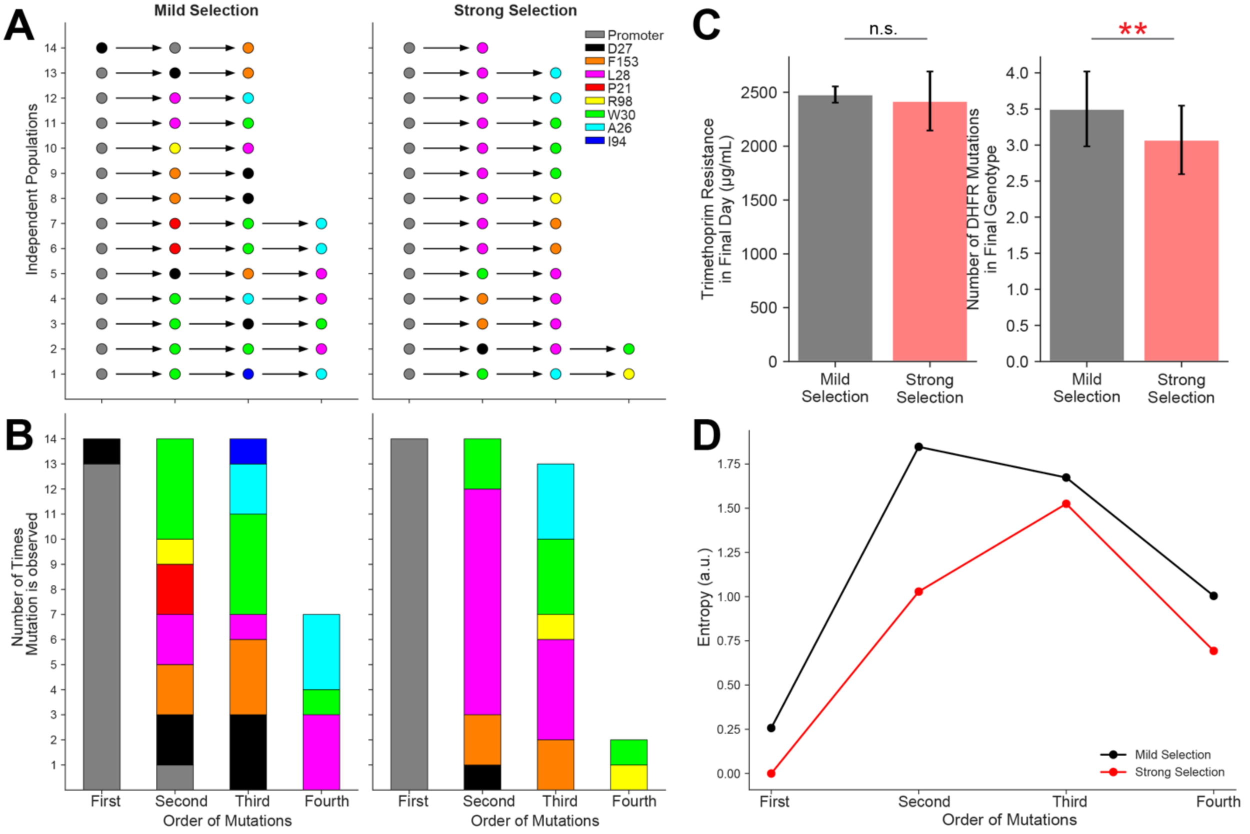

Figure S1: E. coli populations evolved under strong selection had fewer mutations and less genetic diversity compared to populations evolved under mild selection. Morbidostat experiments were run under strong selection (dilution rate of 0.6 h^{-1}) and mild selection (dilution rate of 0.3 h^{-1}). **A)** Genetic trajectories showing the order of DHFR mutations are demonstrated using colored circles. For clarity, only mutational trajectories that survived till the end of morbidostat experiments are shown. Mutations in other subpopulations are listed in Table S7. **B)** Frequencies of mutations in evolved populations. Populations evolved under strong selection had relatively less genetic diversity. The first mutation in the coding region of *folA* was dominantly the L28R mutation (9 times out of 14) under strong selection. In the case of mild selection, the first mutations in the coding region of *folA* were more random. **C)** (left) Under both selection conditions, populations became highly resistant to trimethoprim (MIC ~2.5mg/mL). Final resistance of the populations evolved under strong or mild selection were not significantly different ($p>0.05$, Student t-test). (right) Populations evolving under low dilution rate (mild selection, 0.3 h^{-1}) acquire higher number of mutations (3.5±0.52 mutations vs 3.07±0.47, $p < 0.01$, Student t-test) compared to populations evolving under high dilution rate (strong selection, 0.6 h^{-1}). **D)** We grouped mutations depending on their order of appearance and calculated diversities across evolving populations (as DHFR mutations are selected and fixed) by utilizing Shannon Entropy ($-\Sigma f_i.\log(f_i)$), where f_i is the frequency of each mutation across populations after mutations are grouped according to the order they are acquired. Entropies of populations evolving under mild selection (black line) were consistently higher than the entropy of populations evolving under strong selection (red line).

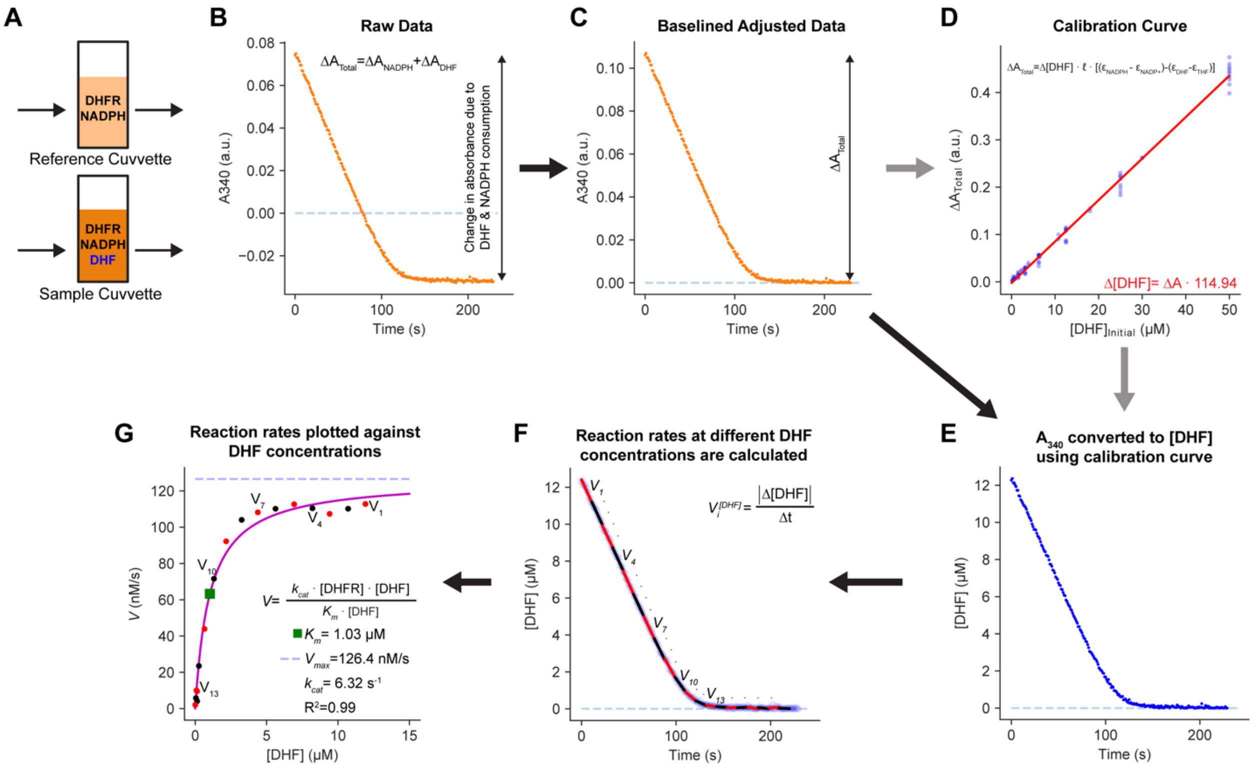

Figure S2: We developed a new spectroscopic assay to rapidly phenotype DHFR variants. (A) All of the spectroscopic measurements were done using a two-cell spectrophotometer from Perkin Elmer (Lambda 650). All experiments were done in MTEN buffer (pH ~ 7) at room temperature (~ 25ºC). In all of the assays, ~20nM of DHFR, 200μM of NADPH, and 12.5μM of Dihydrofolicacid (DHF) were used. For the slow DHFR mutants, 250nM of DHFR was used to speed up the reaction. **(B)** All the absorbance measurements were done at 340 nm by using a reference reaction cuvette that included ~20nM of DHFR and 200μM of NADPH. **(C)** After collecting absorbance data during the reaction, a baseline correction was done such that the final absorption at the end of the reaction was set to 0. **(D)** Next, we performed several experiments using the wild type DHFR and 200μM of NADPH, and measured total absorption change (ΔA_{total}) as a function of initial DHF concentration (ranging from 0 to 50μM). **(E)** Using these values, we calibrated our system and converted A_{340nm} values to DHF concentrations. **(F)** Then, we splitted the reaction curve into several windows (10 to 50 windows, depending on the speed of the mutant DHFR) and calculated the average reaction rates (V_i= |Δ[DHF]/Δt|) and DHF ($[DHF]_i$) concentrations at each window. **(G)** Finally, we plot reaction rates (V_i) against DHF concentrations ($[DHF]_i$) and fit the Michelis-Menten equation to predict k_{cat} and K_m values.

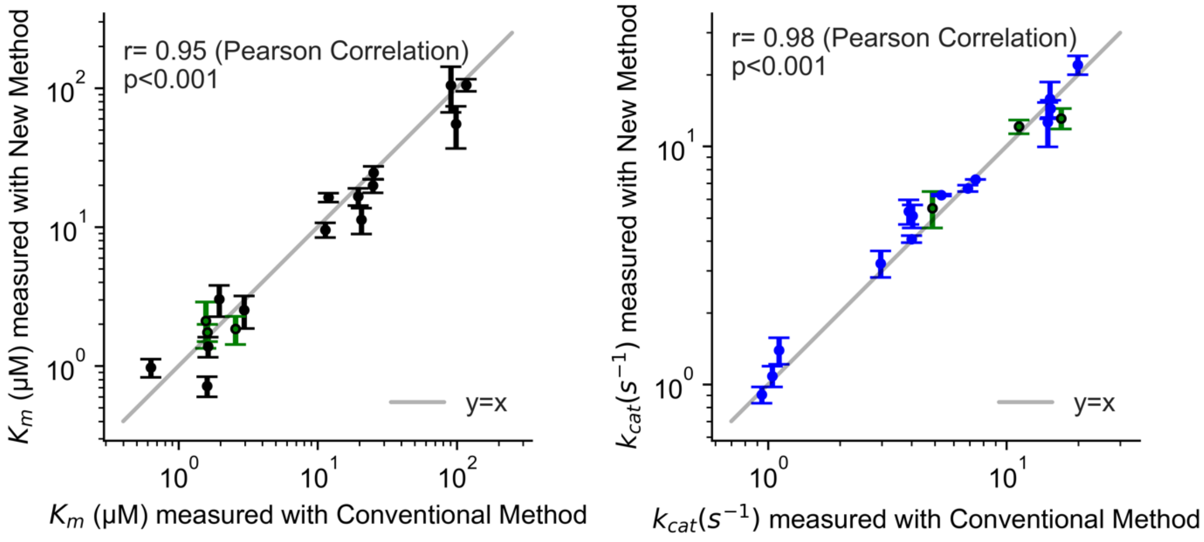

Figure S3: Biochemical properties of DHFR variants extracted by our new spectroscopic assay and the conventional assay are in good agreement. We compared K_m and k_{cat} values of 8 DHFR variants (including the wild-type) coming from 17 matching measurements using our new spectroscopic assay and the conventional method (where initial DHFR activity is monitored at various DHF concentrations in separate experiments). For both K_m (left panel) and k_{cat} (right panel), our new method and the conventional method yielded similar values (r = 0.98 and $p < 10^{-3}$ for k_{cat}; r = 0.98 and $p < 10^{-3}$ for K_m; Pearson Correlation Test).

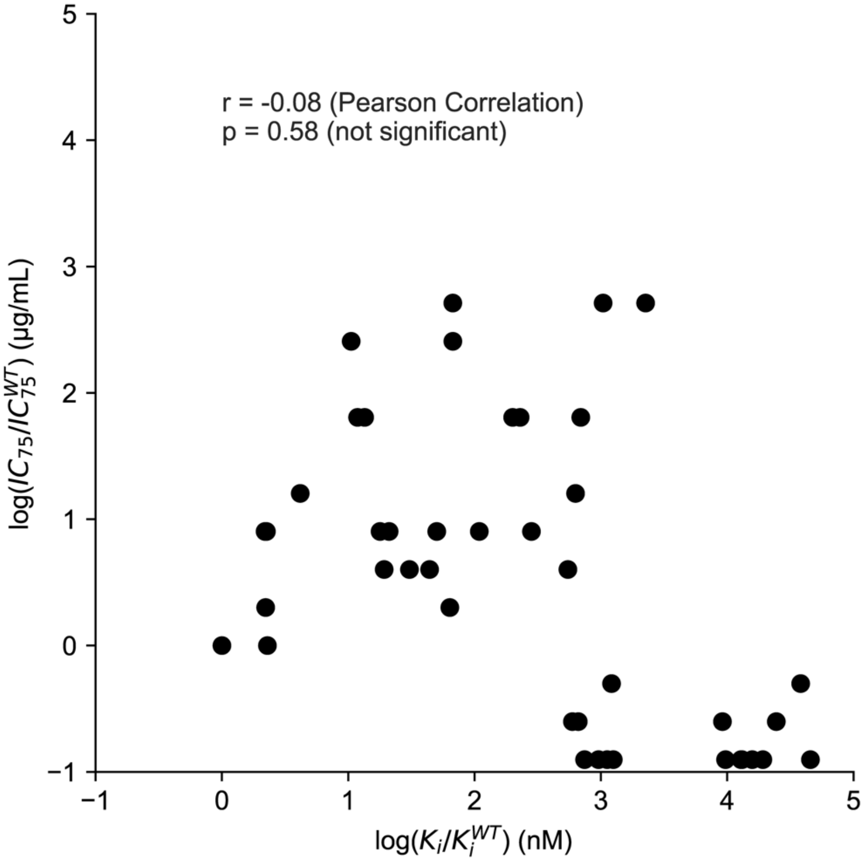

Figure S4: Comparison of K_i and IC_{75} values of 48 DHFR mutants. Black circles represent K_i values of mutant DHFR enzymes (x-axis) and IC_{75} values of bacterial clones with corresponding mutations in the *folA* gene (y-axis). Changes in K_i values of the mutant enzymes by themselves cannot explain resistance changes (IC_{75}) in bacterial clones with corresponding mutations (r = -0.078, p = 0.58, Pearson Correlation Test).

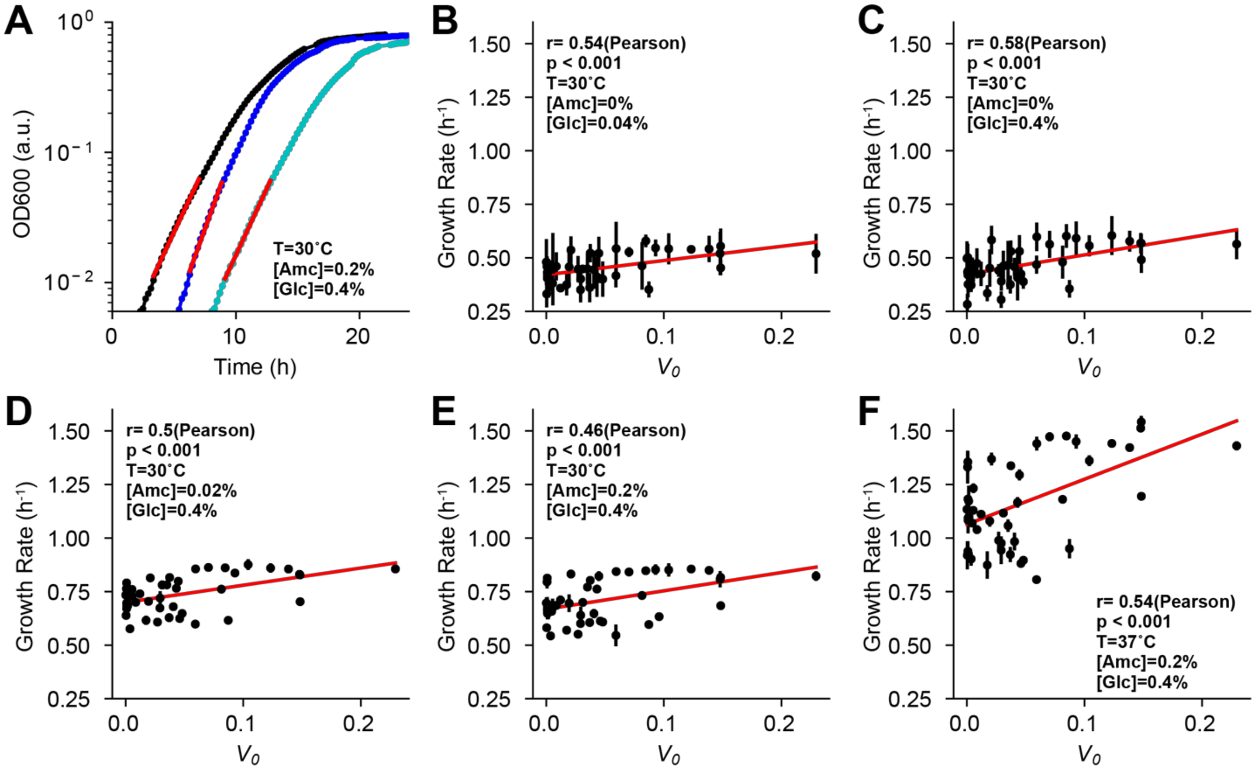

Figure S5: Bacterial growth rates correlate with DHFR's enzymatic activity. A) Growth rates (μ) of *E. coli* cells with DHFR mutations are calculated by fitting an exponential growth function; OD(t) = OD(0). $e^{\mu.t}$, to the cell density (OD600) readings. **B-F)** Mean growth rate values (± standard deviation) of all mutations are measured for different M9 minimal media compositions and temperature (*T*). Correlation between V_0 and growth rate is calculated using Pearson Correlation test. *r*: correlation coefficient, *p*: significance. [Amc] stands for amicase concentration; [Glc] stands for glucose concentration.

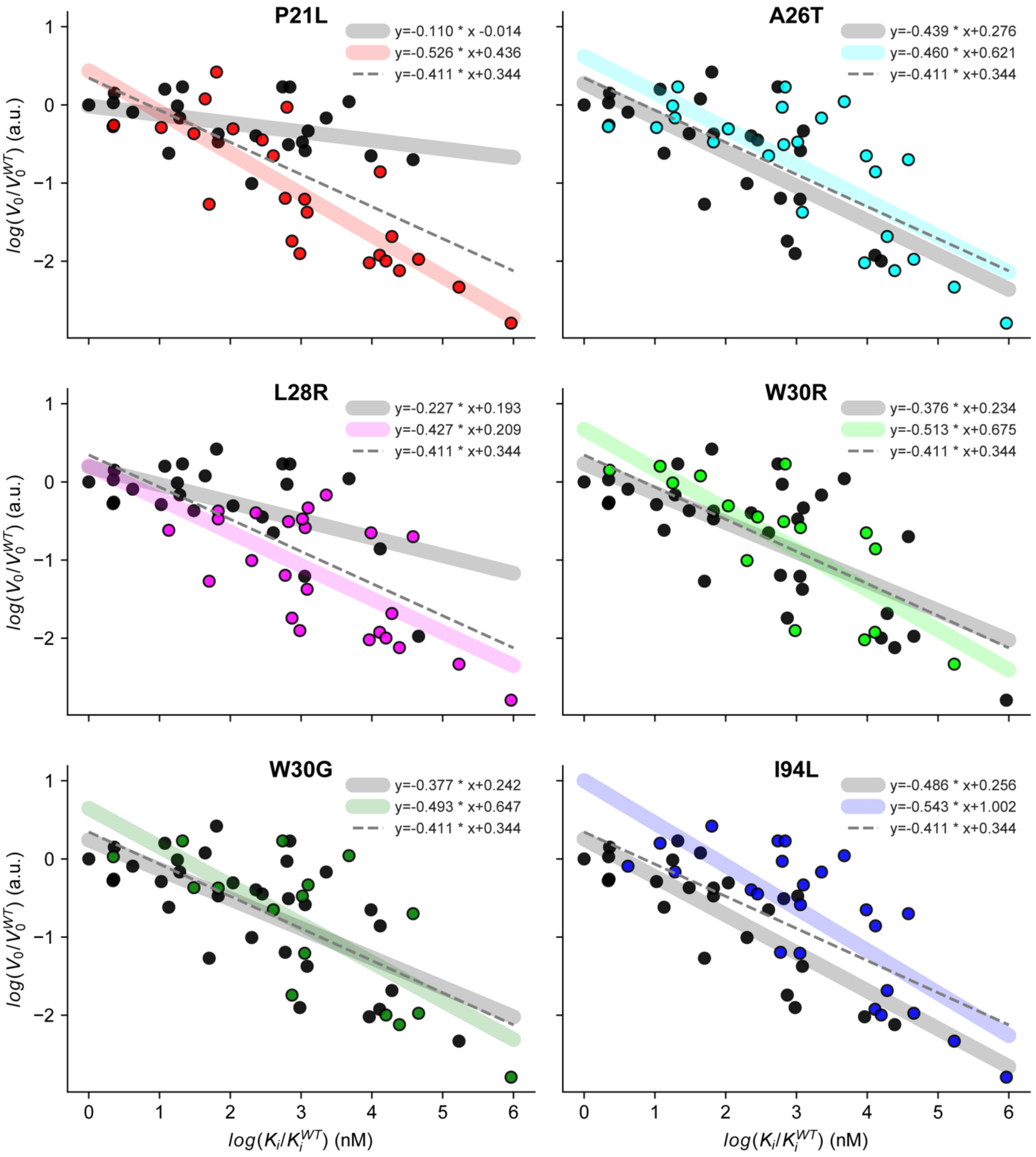

Figure S6: Bifurcation in the V_0-K_i plane can only be explained by the P21L mutation. We grouped the mutants depending on having or missing a particular mutation and tested whether the trend of the two groups differed. Colored and black circles are used for the genotypes with or without a particular mutation (i.e. cyan for A26T), respectively. Colored and gray lines are used to display trends in the V_0-K_i plane with and without a particular mutation, respectively. Dashed line is used to display the overall trend when all 48 genotypes are included.

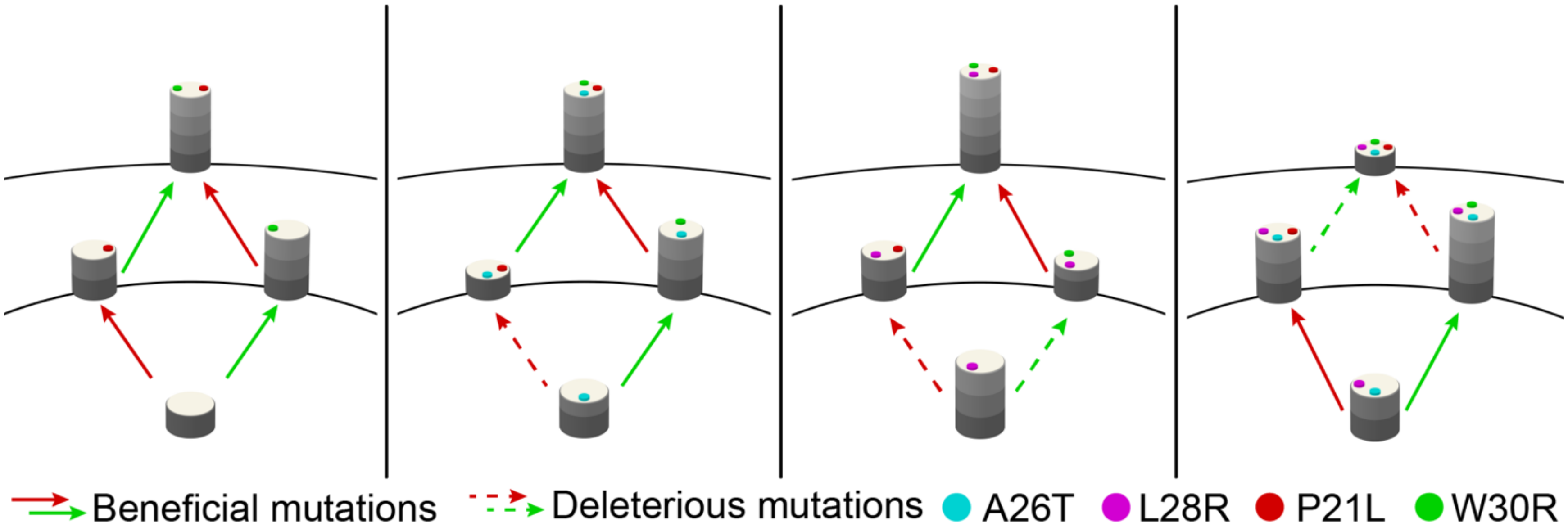

Figure S7: Fitness effects of mutations are context dependent in the presence of high-order epistasis. Illustration of how individual and combined effects of two beneficial mutations (red and green filled circles) change on (top left panel) the wild-type genetic background; (top right and bottom left panels) on genetic backgrounds carrying a single beneficial mutation (cyan or magenta filled circle); and (right bottom panel) on a genetic background with two beneficial mutations. In such a complicated scenario because of high-order epistasis, it is not possible to predict fitness of genotypes with multiple mutations by knowing fitness effects of single mutations and pairwise interactions. Colored circles on the surface of cylinders represent mutations. Height of cylinders represent fitness. Solid arrowed lines and dashed arrowed lines represent beneficial and deleterious mutations, respectively.

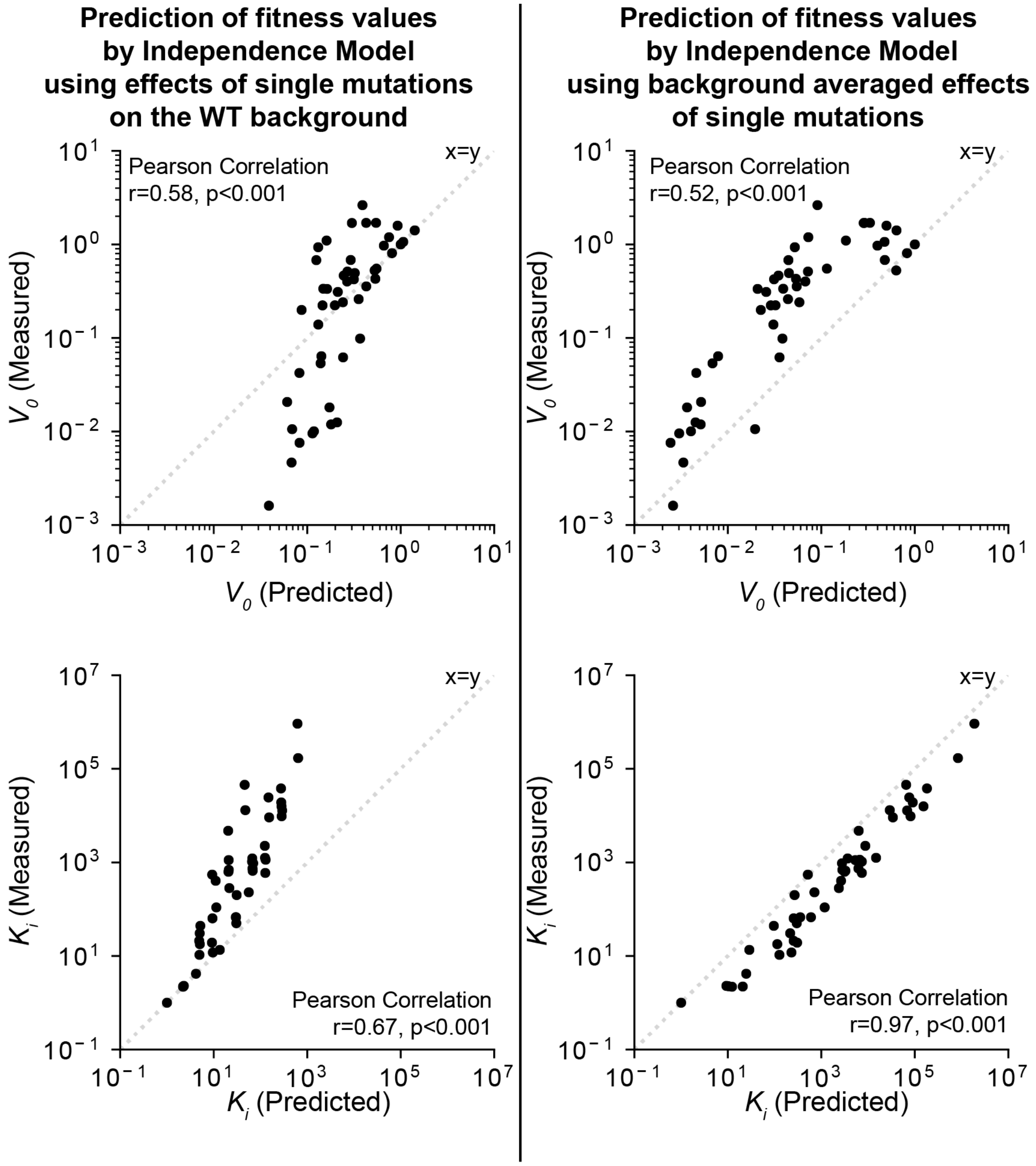

Figure S8: Comparison of predicted V_0 and K_i values using additivity with experimentally measured values. Panels on left side shows x-axis values predicted with an independence model using single mutant data. Panels on the right shows x-axis values predicted with an Independence model using the (geometric) mean effects of single mutants.

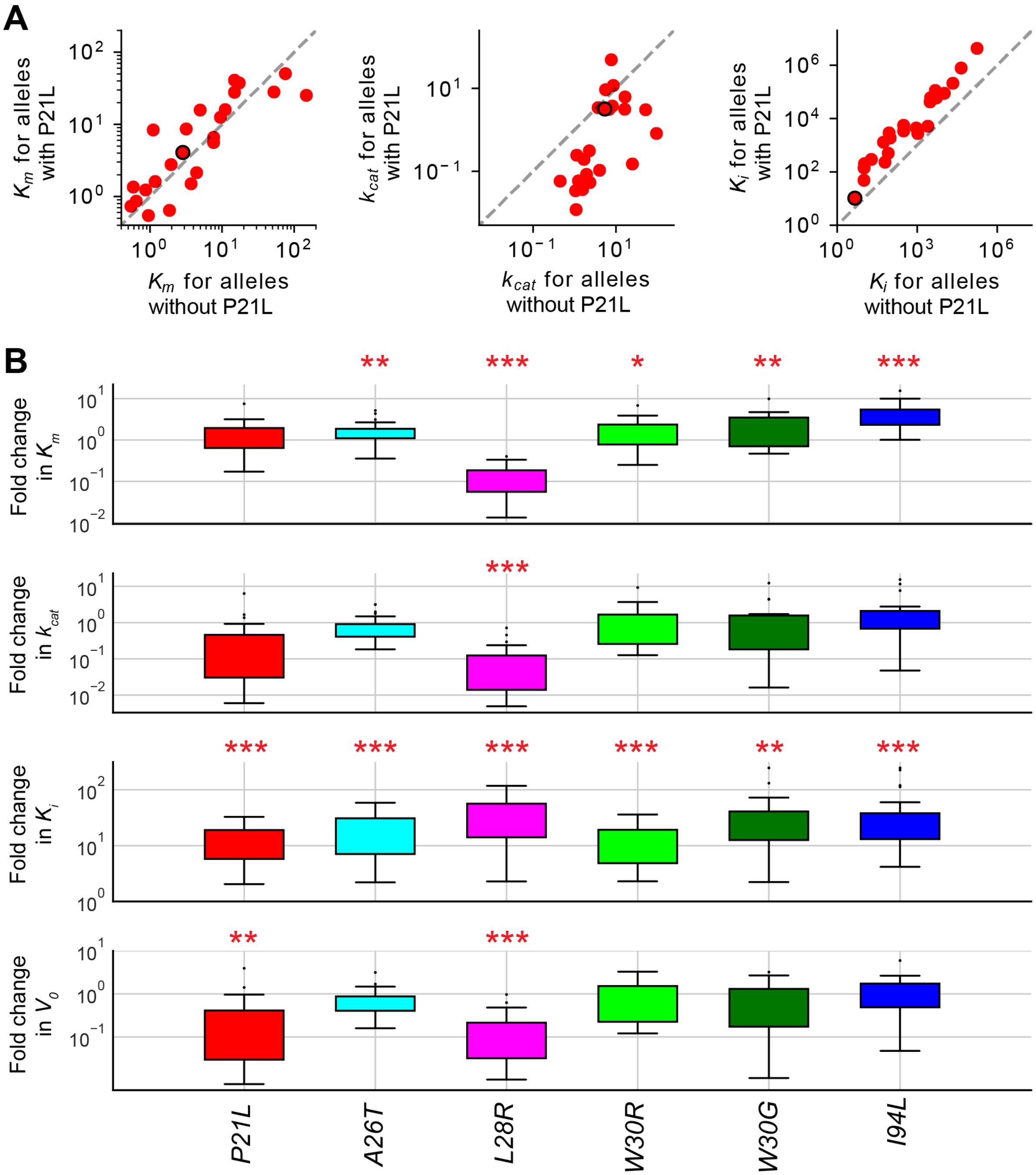

Figure S9: Mean effects of DHFR mutations in catalytic activity and trimethoprim binding. A) Each marker in upper panels show fitness changes when a mutant acquires P21L mutation. x axis shows K_m, k_{cat} and K_i values of mutant alleles without P21L mutation and y axis shows the values mutant alleles with P21L mutation. For instance, the black encircled points have the K_m, k_{cat} or K_i value of WT on x axis and corresponding values for P21L on y axis. **B)** Fold change effects when each single mutant is added on top of all other genotypes. Briefly, fitness change due to a particular mutation (i.e. effect of L28R in the background of the genotype carrying W30R and I94L mutations; fold change in K_m = $K_m^{(L28R+W30R+I94L)}$/ $K_m^{(W30R+I94L)}$) is calculated by computing the geometric mean of fold changes on all possible genetic backgrounds. Student's t-test (two tailed) is used to quantify significance of K_m, k_{cat} and K_i, V_0 changes relative to the wild type DHFR (*: p<0.05; **: p<0.01; ***: p<0.001).

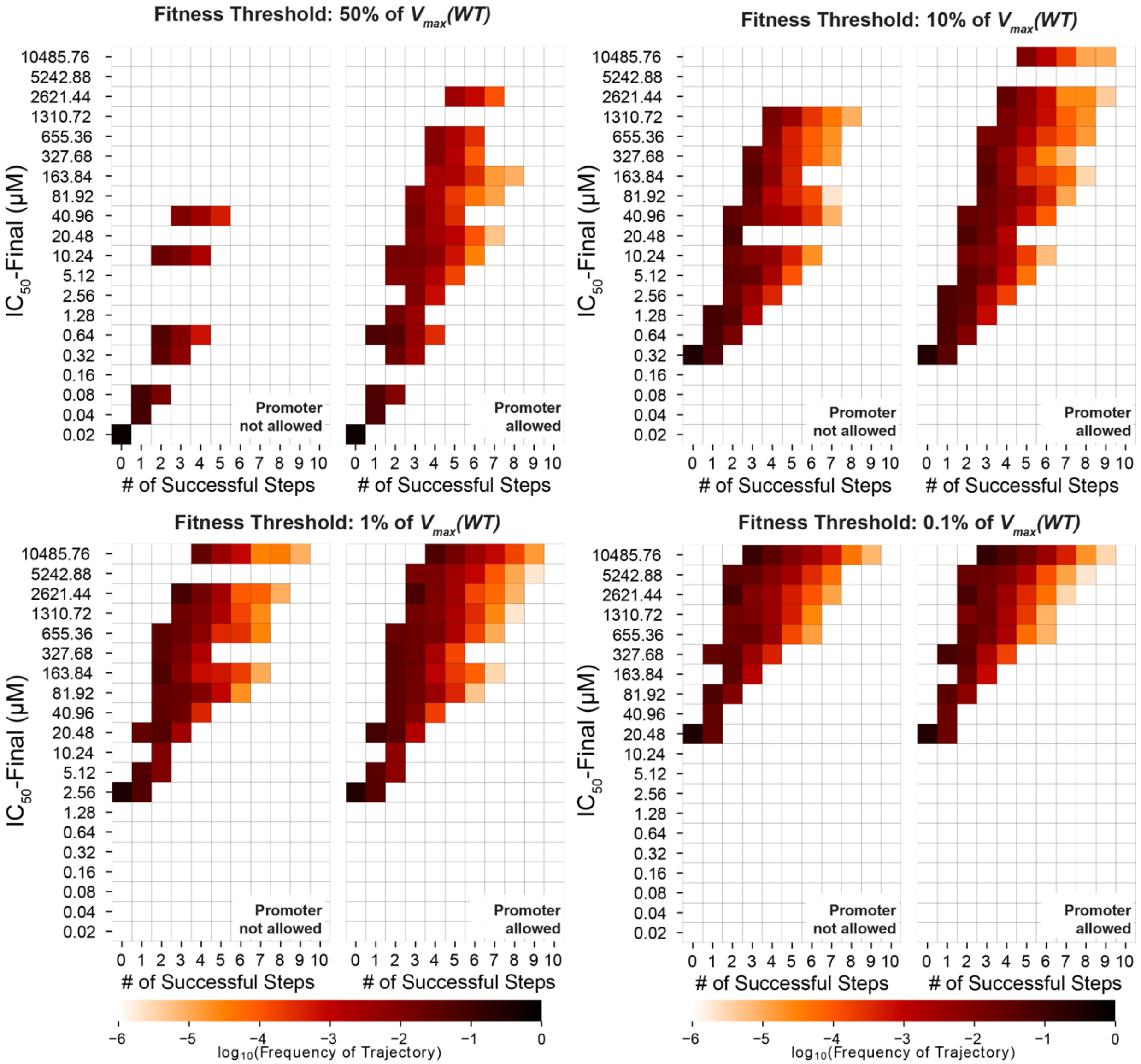

Figure S10: Simulations are repeated for different threshold values (%V_0^{WT} as threshold) showing the number and length of evolutionary trajectories that reach to fitness peaks drastically increase if minimum fitness thresholds are assumed to be lower.

Captions for Supplementary Tables

Table S1: Illumina (HiSeq) whole genome sequencing analysis summary for the *E. coli* mutants isolated from cultures evolved in the morbidostat. These mutants were isolated from the final day of the morbidostat experiments.

Table S2: Validation of our new *in vitro* assay for quantifying catalytic efficiency of DHFR. K_m and k_{cat} values of eight DHFR mutants were measured multiple times using the conventional method and our new method. This data set is displayed in Figure S3.

Table S3: K_m, k_{cat}, and K_i values of eleven DHFR mutants with single amino acid replacements (Figure 2) are provided.

Table S4: Experimentally measured K_m, k_{cat}, and K_i values for 48 DHFR mutants that include all possible combinations of P21L, A26T, L28R, W30G, W30R, and I94L mutations.

Table S5: Background-averaged fitness effects (on K_m, k_{cat}, and K_i values) of P21L, A26T, L28R, W30G, W30R, and I94L mutations. This data is partially displayed in Figure S9.

Table S6: Summary analysis of the Gillespie simulations for predicting viable evolutionary trajectories.

Table S7: Frequency of DHFR mutations in the morbidostat experiments are shown. For every mutation listed, we calculated frequency of appearance as the first mutation in the coding region of DHFR as well as the frequency of fixation in the population.
